# Study of Superbase-Based Deep Eutectic Solvents as the Catalyst in the Chemical Fixation of CO_2_ into Cyclic Carbonates under Mild Conditions

**DOI:** 10.3390/ma10070759

**Published:** 2017-07-07

**Authors:** Sara García-Argüelles, Maria Luisa Ferrer, Marta Iglesias, Francisco Del Monte, María Concepción Gutiérrez

**Affiliations:** 1 Materials Science Factory, Instituto de Ciencia de Materiales de Madrid (ICMM), Consejo Superior de Investigaciones Científicas (CSIC), Campus de Cantoblanco, C/Sor Juana Inés de la Cruz 3, 28049 Madrid, Spain; sara_g.arguelles@yahoo.es (S.G.A.); marta.iglesias@icmm.csic.es (M.I.); delmonte@icmm.csic.es (F.D.M.); 2 Departamento de Tecnología Química y Energética, Tecnologia Química y Ambiental y Tecnología Mecánica y Química Analítica, Universidad Rey Juan Carlos, 28933 Madrid, Spain

**Keywords:** eutectic solvents, CO_2_ absorption, CO_2_ fixation, superbases

## Abstract

Superbases have shown high performance as catalysts in the chemical fixation of CO_2_ to epoxides. The proposed reaction mechanism typically assumes the formation of a superbase, the CO_2_ adduct as the intermediate, most likely because of the well-known affinity between superbases and CO_2_, i.e., superbases have actually proven quite effective for CO_2_ absorption. In this latter use, concerns about the chemical stability upon successive absorption-desorption cycles also merits attention when using superbases as catalysts. In this work, ^1^H NMR spectroscopy was used to get further insights about (1) whether a superbase, the CO_2_ adduct, is formed as an intermediate and (2) the chemical stability of the catalyst after reaction. For this purpose, we proposed as a model system the chemical fixation of CO_2_ to epichlorohydrin (EP) using a deep eutectic solvent (DES) composed of a superbase, e.g., 2,3,4,6,7,8-hexahydro-1*H*-pyrimido[1,2-*a*]pyrimidine (TBD) or 2,3,4,6,7,8,9,10-octahydropyrimido[1,2-*a*]azepine (DBU), as a hydrogen acceptor and an alcohol as a hydrogen bond donor, e.g., benzyl alcohol (BA), ethylene glycol (EG), and methyldiethanolamine (MDEA), as the catalyst. The resulting carbonate was obtained with yields above 90% and selectivities approaching 100% after only two hours of reaction in pseudo-mild reaction conditions, e.g., 1.2 bars and 100 °C, and after 20 h if the reaction conditions of choice were even milder, e.g., 1.2 bars and 50 °C. These results were in agreement with previous works using bifunctional catalytic systems composed of a superbase and a hydrogen bond donor (HBD) also reporting good yields and selectivities, thus confirming the suitability of our choice to perform this study.

## 1. Introduction

The expected decrease of global oil supplies makes necessary the search for new precursors for plastic materials offering an alternative to petroleum-based feedstock [1]. CO_2_ coupling to epoxides for carbonates production is attracting increased attention because it is of interest by two-fold [2,3]. The first reason is of the environmental type and considers captured CO_2_ as a feedstock in the chemical industry for preventing the extremely high CO_2_ emission resulting every year from the continuous use of fossil fuels for combustion, e.g., 32.3 billion tons only in 2014 [4]. Second, cyclic carbonates can be used as precursors of some of the most interesting synthetic polymers that society is nowadays demanding, e.g., polycarbonates, used as electrolytes in batteries and supercapacitors in portable electronic devices [5,6]. Based on these reasons, developing systems based on CO_2_ capture and storage that afterwards are also capable of chemical fixation is an attractive approach, i.e., in terms of both environmental protection and resource utilization, to obtain value-added organic chemicals [7].

Quaternary ammonium [8] and phosphonium salts [9], including superbases like 2,3,4,6,7,8-hexahydro-1*H*-pyrimido[1,2-*a*]pyrimidine (TBD) [10] or 2,3,4,6,7,8,9,10-octahydropyrimido[1,2-*a*]azepine (DBU) [11], alkali metal halides [12], ionic liquids (ILs) [13], metalloporphyrin [14] or metallosalen complexes [15] and N-heterocyclic carbenes [16], have been widely used as catalysts to activate CO_2_ and thus facilitate its reaction with an epoxide. Among them, metal-free systems are indeed the most interesting ones in environmental terms [17]. Moreover, most of these processes combined the use of high gas pressures (typically above 10 bars and occasionally up to 100 bars), high reactor temperatures (typically above 120 °C and occasionally up to 160 °C) and/or long reaction times (typically above 6 h, and occasionally up to 24 h) [7,18], whereas milder condition reactions, helping to ameliorate certain limitations in terms of both cost and safety, are indeed required if one desires to scale up the process. Interestingly, only a few reports have described syntheses carried out under mild reaction conditions. For instance, Wang et al. have reported about yields of up to 86% in the cycloaddition of CO_2_ to styrene oxide using a catalytic amount of benzyl halide and a small excess amount of N,N-dimethylformamide for reactions carried out over 24 h at 1 bar and 120 °C [19]. In terms of mildness, further improvement was provided by Liu et al. who, performing also the cycloaddition of CO_2_ to styrene oxide, obtained yields of up to 93% using CaBr_2_ as the catalyst and DBU as the co-catalyst for reactions carried out over 12 h at 1 bar and 100 °C [20]. Finally, Wang et al. have demonstrated that the cycloaddition of CO_2_ to epichlorohydrin takes place with high-to-excellent yields, e.g., up to 97%, when the reaction was carried out over 24 h at 1 bar and 25 °C, and *n*Bu_4_NI was used as the co-catalyst in combination with different hydrogen bond donors (HBDs) [21]. With regard to this latter case, it is widely accepted that the co-existence of HBDs and ammonium salts activates the reaction for the CO_2_ fixation to the epoxide thanks to the characteristic synergistic effect exhibited by bifunctional catalysts [22,23,24,25]. Actually, bifunctional catalysts of different types are attracting much attention not only in cycloadditions of CO_2_ to styrene oxide [8,26,27], but also in many other different reactions [28,29,30,31].

Within the context of using HBDs and ammonium salts as bifunctional catalysts, deep eutectic solvents (DESs) appear as a promising alternative. DESs, first described by Abbott and co-workers [32], are molecular complexes typically formed between quaternary ammonium salts and HBDs. More recently, eutectics with a particular composition and/or physico-chemical behavior have received different names, e.g., low-melting eutectic mixtures of sugar, urea and salts first described by König and co-workers [33]; natural deep eutectic solvents (NADES) by Choi et al. [34]; and low-transition temperature mixtures (LTTMs) by Kroon and co-workers [35]. Actually, DESs have been used for CO_2_ absorption, first just as solvents of alkylamines typically used as CO_2_ absorbents [36] and then designing DESs with intrinsic CO_2_ absorption capabilities [37,38,39,40,41,42,43,44]. Interestingly, [urea-Zn]I_2_ eutectic-based ILs have recently been used to catalyze the cycloaddition of CO_2_ to propylene oxide finding yields of up to 95% for reactions carried out over 2 h at 10 bars and 120 °C [45]. Moreover, DES-based bifunctional catalysts formed between ILs and a co-catalysts have also provided excellent figures of conversion and selectivity under mild reaction conditions [46].

Herein, we aim to use DESs composed of a superbase, e.g., TBD and DBU, and different HBDs, benzyl alcohol (BA), ethylene glycol (EG), and methyldiethanolamine (MDEA), as the bifunctional catalyst for the chemical fixation of CO_2_ into epichlorohydrin (EP, also known as 2-chloromethyl oxirane). Our group has described different polymerizations where DESs act as all-in-one solvent-template-reactant systems, i.e., DESs play the role of precursor, template, and reactant medium [47]. DESs have shown a high capability to capture CO_2_ [48,49]. Also interesting for the purpose of the current work was the use of a DES composed of TBD and methanesulfonic acid as the bifunctional catalyst in the ring-opening polymerization (ROP) of ε-caprolactone [50] based on the general assumption that H-bond-complex-like intermediates play a critical role activating ROPs [51,52]. In this case, we have explored the use of not only TBD, but also DBU, and the reasons were two-fold; (1) both TBD and DBU, typically as ILs, have exhibited high CO_2_ absorption capabilities [53,54,55,56] and (2) both TBD and DBU, either as ILs or by themselves, have exhibited an excellent performance as catalysts in the CO_2_ fixation to epoxides [11,20,57,58,59,60,61]. Thus, we first characterized the DESs mainly with regard to their performance for CO_2_ capture. Afterwards, we evaluated the performance of the most suitable one as the catalyst for CO_2_ fixation to EP in experimental conditions of pressure, temperature and reaction time, e.g., 1.2 bars, 100 °C and 2 h and 1.2 bars, 50 °C and 20 h, which overall, could be considered as one of the mildest ones ever studied using superbases as catalysts. Moreover, we performed an intensive study of reagents, products and intermediates by ^1^H and ^13^C NMR spectroscopy so that, based on it, we proposed a plausible reaction mechanism. Eventually, this study also illustrates the poor chemical stability of superbases in both their bare and DES form, as anticipated in previous works using superbases for CO_2_ absorption [62].

## 2. Results and Discussion

As mentioned in the experimental part, we prepared DESs composed of DBU and TBD as the ammonium salts and ethylene glycol (EG), benzyl alcohol (BA) and methyldiethanolamine (MDEA) as the HBDs (Figure S1). In particular, we prepared a set of ten DESs; these were DBU:BA and TBD:BA with molar ratios of 1:1 and 1:4, DBU:EG and TBD:EG with molar ratios of 1:1 and 1:4 and DBU:MDEA and TBD:MDEA with a molar ratio of 1:2, e.g., DBU(1):BA(1), TBD(1):BA(1), DBU(1):BA(4), TBD(1):BA(4), DBU(1):EG(1), TBD(1):EG(1), DBU(1):EG(4), TBD(1):EG(4), DBU(1):MDEA(2) and TBD(1):MDEA(2). 

**Figure 1:**
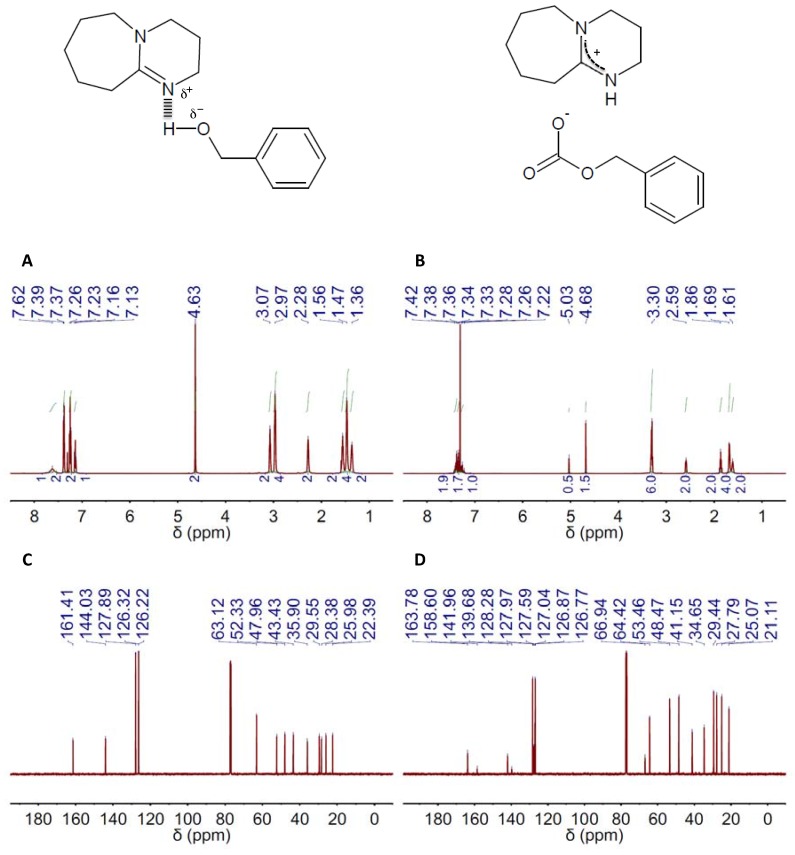
^1^H and ^13^C NMR spectra (left and right columns, respectively) of the deep eutectic solvent (DES) composed of 2,3,4,6,7,8,9,10-octahydropyrimido[1,2-*a*]azepine (DBU) and benzyl alcohol (BA) in a 1:1 molar ratio, e.g., DBU(1):BA(1), before (**A**,**C**) and after (**B**,**D**) CO_2_ absorption. The chemical structures of both the DES resulting from H-bond interaction between DBU and BA in the DES and the salt formed upon CO_2_ absorption on DES are also included.

**Figure 2:**
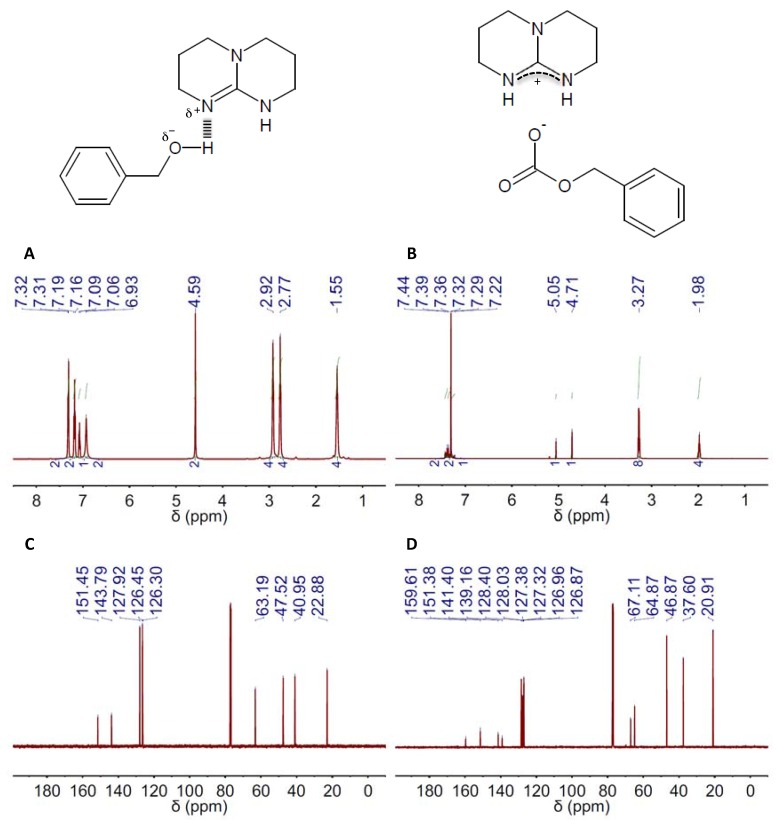
^1^H and ^13^C NMR spectra (left and right columns, respectively) of the DES composed of 2,3,4,6,7,8-hexahydro-1*H*-pyrimido[1,2-*a*]pyrimidine (TBD) and BA in a 1:1 molar ratio, e.g., TBD(1):BA(1), before (**A**,**C**) and after (**B**,**D**) CO_2_ absorption. The chemical structures of both the DES resulting from H-bond interaction between TBD and BA in the DES and the salt formed upon CO_2_ absorption on DES are also included.

The ^1^H NMR spectra of the different DESs revealed the formation of H-bond complexes, i.e., the signals of the components were shifted to upper chemical fields as compared to those of the individual components (see DBU(1):BA(1) and TBD(1):BA(1) in, respectively, Figure 1A and Figure 2A, and Tables S1 and S2; and the remaining DESs and their respective individual components in Figures S2–S9 and Tables S3–S6 in the Supporting Information). The ^13^C NMR spectra of the different DESs were also obtained (see Figure 1C and Figure 2C, Tables S7 and S8, Figures S2–S9 and Tables S9–S12).

The capacity of TBD- and DBU-based DESs for CO_2_ absorption was studied by both ^1^H and ^13^C NMR spectroscopy. Carbonate formation was the absorption mechanism proposed by Jessop and coworkers for mixtures of superbases and alcohols, so ultimately, one obtain a salt where the carbonate resulting from CO_2_ fixation to the hydroxyl group is the anion and the protonated form of the superbase the cation (Figure 1 and Figure 2, Figure S10) [63]. CO_2_ absorption by these means was actually reflected in both the ^1^H and ^13^C NMR spectra of both TBD and DBU (Figure 1B,D and Figure 2B,D, Figures S2–S9 and Tables S1–S12). The most evident signature of the formation of a carbonate moiety was the appearance of a peak at ca. 158 ppm in the ^13^C NMR spectra (Figure 1D and Figure 2D, Figures S2–S9 and Tables S7–S12). CO_2_ chemisorption was also evident in the peak positions in both the ^1^H and ^13^C NMR spectra of every HBD, e.g., BA, EG and MDEA, because of the conversion of hydroxyl moieties into carbonate ones (Figure 1B,D and Figure 2B,D, Figures S2–S9 and Tables S1–S12). Actually, the ratio between the intensity of the original peak, e.g., methylene groups in alpha position to hydroxyl, and the new one, e.g., methylene groups in alpha position to carbonate, will allow estimating the CO_2_ absorption capacity of the different DESs. Thus, a fully-efficient CO_2_ absorption process in either DBU(1):BA(1) or TBD(1):BA(1) would result in the complete conversion of hydroxyl moieties into carbonate ones, whereas both hydroxyl and carbonate moieties would coexist in any process where efficiency is eventually below 100%. This latter was actually the situation for DBU(1):BA(1) and TBD(1):BA(1) where CO_2_ absorption promoted a significant viscosity increase that ultimately impeded gas diffusion throughout the entire volume. Under these circumstances, the presence of both hydroxyl and carbonate moieties after CO_2_ absorption will duplicate the most significant peaks in the ^13^C NMR spectra. For instance, peaks at ca. 128.0, 126.8 and 127.0 ppm assigned respectively to C3&C5, C4, and C2&C6 in the carbonated form of DBU(1):BA(1) appeared besides those at 128.3, 127.0 and 126.9 ppm assigned to the same carbons in the non-carbonated form (see Table S7), and a similar trend was observed for TBD(1):BA(1) (see Table S8). Duplication also occurred in the ^1^H NMR spectra for the peak assigned to methylene groups in alpha position to the hydroxyl; see, for instance, the peaks at 4.63 ppm for DBU(1):BA(1) and at 4.68 and 5.03 ppm for DBU(1):BA(1) after CO_2_ absorption in Figure 1A,B, and Table S1. 

It is worth noting that ratios between the intensity of the original peak, e.g., methylene groups in alpha position to the hydroxyl, and the new one, e.g., methylene groups in alpha position to the carbonate, approaching the maximum-absorption-capability of these mixtures of superbases and alcohols, e.g., one, based on the above-described absorption mechanism, were obtained in DBU- and TBD-based DESs formed with an excess of alcohol, e.g., molar ratios of 1:4, in agreement with the above-described dependence between efficiency and viscosity (Table 1). Interestingly, the peak positions at the ^13^C NMR spectra of the amidinium carbon also reflected the improvement of the absorption capability in both DBU- and TBD-based DESs formed with an excess of alcohol, i.e., the larger the number of carbonate moieties, the more deshielded the position of the peak because of the overall increase of protonated superbase molecules. 

After determining the CO_2_ absorption capacity of our DESs, we next studied their catalytic activity in the chemical fixation of CO_2_. As mentioned in the Introduction, the use of DBU and TBD either by themselves or in combination with HBDs for chemical fixation of CO_2_ into epoxides has been studied in several works [20,58,59,60,61,62,63]. In our case, we also started studying the catalytic performance of DBU and TBD by themselves to, afterwards, compare this performance with that provided by their respective DESs. The reaction of choice was one of the most widely studied by different authors, e.g., the fixation of CO_2_ into EC, because our purpose was studying the effect of various parameters on the reaction outcome and to contextualize our results with those previously reported.

**Figure 3:**
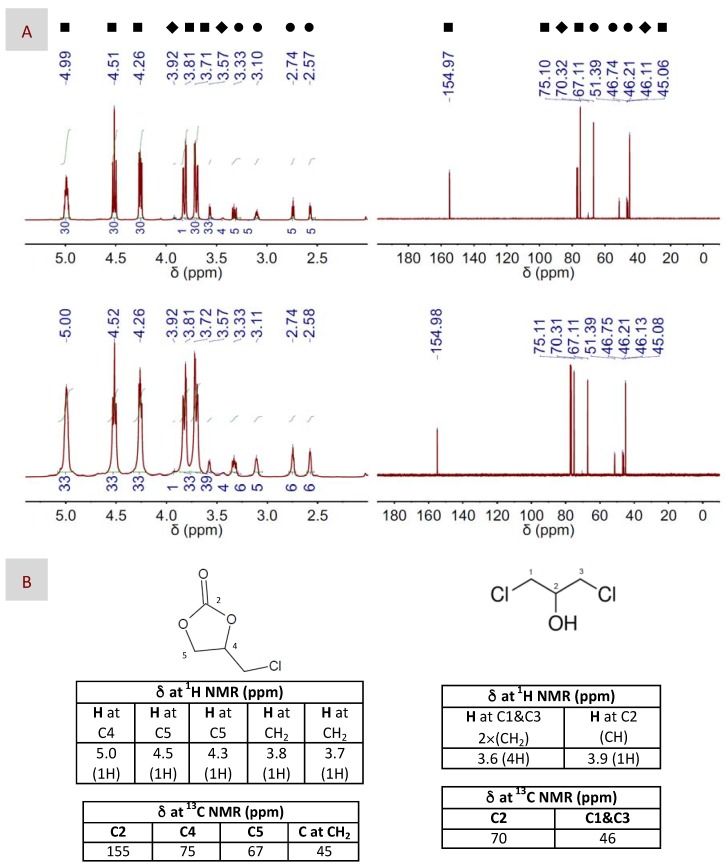
(**A**) ^1^H and ^13^C NMR spectra (left and right columns, respectively) of both the main product, e.g., 4-(chloromethyl)-1,3-dioxolan-2-one, and the sub-product, e.g., 1,3-dichloropropan-2-ol, resulting after CO_2_ fixation to EP using either DBU (top panel) or DBU(1):BA(1) DES (bottom panel) as the catalyst. The catalyst to EP molar ratio was 1:100. Symbols assign peaks to their respective molecules, i.e., ■ for 4-(chloromethyl)-1,3-dioxolan-2-one, ♦ for 1,3-dichloropropan-2-ol and ● for EP. (**B**) Chemical structures of 4-(chloromethyl)-1,3-dioxolan-2-one and 1,3-dichloropropan-2-ol, as well as chemical shifts assigned to their respective peaks at the ^1^H and ^13^C NMR spectra. The reaction conditions were 100 °C, 1.2 bars and 2 h for these particular spectra, but similar spectra were obtained in the different reaction conditions described in Table 2.

Thus, we first studied DBU as the only catalyst in experimental conditions, e.g., 100 °C, 6 bars and 1–2 h (see Entries 1 and 2 in Table 2), that were similar to or slightly milder than those described in previous works also using bare DBU. The product resulting after reaction, e.g., 4-(chloromethyl)-1,3-dioxolan-2-one, was identified by ^1^H and ^13^C NMR spectroscopy. In particular, we followed how the peaks of EP in both the ^1^H and the ^13^C NMR spectra, i.e., at ca. 2.6, 2.7, 3.1, 3.4 and 3.6 ppm and at ca. 46–47 and 51 ppm, respectively (Figure S1), vanished, partially or completely, depending on the yield, with the appearance of peaks assigned to 4-(chloromethyl)-1,3-dioxolan-2-one, i.e., at ca. 3.7, 3.8, 4.3, 4.5 and 5.0 ppm and at ca. 45, 67, 75 and 155 ppm, respectively (Figure 3). GC and ^1^H NMR spectroscopy were used to determine the catalytic activity in terms of both yield and selectivity; see the experimental part for further details. In this regard, our results were in agreement with those described before, with yields of nearly 70% after 1 h of reaction and slightly above 80% after 2 h. Our next experiment was carried out in milder conditions by decreasing the pressure used in the reactor from 6 down to 1.2 bars; see Entries 3 and 4 in Table 2. Interestingly, we found that the catalytic efficiency of DBU at low-pressure-conditions was basically identical to, or even slightly above, that at high ones. Meanwhile, decreasing the reaction temperature resulted in lower yields, the recovery of which required the combination of longer reaction times and an increased catalyst concentration, e.g., 20 h and 10:100; see Entries 5–8 in Table 2. TBD exhibited a quite similar performance to DBU when used as the only catalyst; see Entries 9–13 in Table 2. The selectivity was around 98–99% in every case. Besides 4-(chloromethyl)-1,3-dioxolan-2-one, the second product obtained in a minor fraction was identified by ^1^H and ^13^C NMR spectroscopy as 1,3-dichloropropan-2-ol, i.e., see the peaks at ca. 3.6 and 3.9 ppm in the ^1^H spectrum and at ca. 46 and 70 ppm in the ^13^C NMR spectrum depicted in Figure 3.

As mentioned in the Introduction, different reports have demonstrated that the use of bifunctional catalysts composed of ammonium salts and HBDs as co-catalysts proved effective in catalytic terms. Thus, our next objective was elucidating whether this was also the case when DESs composed of a superbase, e.g., DBU, and an HBD, e.g., EG, BA or MDEA, were used as catalysts; see Entries 14–25 in Table 2. As for bare DBU, we first studied the CO_2_ fixation into EP at 100 °C and 6 bars, over 1 or 2 h, and with a molar ratio of catalysts versus EP of 1:100. We just used as catalysts the 1:1 molar ratio of BA- and EG-based DESs because DES viscosity was not a problem in the reaction conditions, i.e., DESs dissolved in EP rather in their neat form. No significant differences in yields and selectivities were found between bare DBU and both EG- and BA-based DESs. However, the MDEA-based DES indeed exhibited enhanced yields, with figures that approached 80% after 1 h of reaction and up to 90% after 2 h. In all of these DES-based cases, the use of lower pressures, e.g., 1.2 versus 6 bars, improved the catalytic efficiency in a similar fashion as that described above for bare DBU. 

TBD-based DESs, e.g., TBD(1):BA(1), TBD(1):EG(1) and TBD(1):MDEA(2), were studied at the mildest experimental conditions where DBU-based ones exhibited good catalytic performances, that is 100 °C, 1.2 bars and 2 h; see Entries 26–28 in Table 2. The pattern found for these DESs was similar to that described above for DBU, that is TBD-based DESs worked slightly better that bare TBD. Meanwhile, smaller differences were found among TBD-based DESs with different HBDs than among DBU-based DESs with different HBDs; compare, respectively, Entries 26, 27 and 28 with 17, 21 and 25 in Table 2.

We finally evaluated the catalytic performance of DESs under milder conditions, that is by decreasing the reaction temperature to 50 °C. In this particular case and just focusing on BA-based DESs, we found that yields experienced a dramatic decrease (more so for TBD- than for DBU-based DESs; compare Entries 5, 9, 29 and 33 in Table 2) for reactions carried out at 1.2 bars and over 2 h. As for bare DBU and TBD, a combination of longer reaction times and increased catalyst concentrations (see Entries 30–32 and 34–36 in Table 2) was required to finally recover yields approaching 100%, e.g., for 10:100 and 20 h of reaction.

In view of these results, we could conclude that the catalytic performance found in this work for both DBU- and TBD-based DESs was in the range of the best data recently reported for similar co-catalytic systems. For instance, Liu et al. have recently reported yields of up to 84% with selectivities of up to 91% using co-catalytic systems composed of calcium salts, e.g., CaBr_2_, CaCl_2_, CaI_2_ or CaF_2_, and either TBD or DBU when the fixation of CO_2_ into EP was carried out at 100 °C, 1.0 bars and over 12 h [20], whereas, in our case and for similar experimental conditions, e.g., 100 °C, 1.2 bars, yields above 90% and selectivities approaching 100% were obtained in just 2 h of reaction. Moreover, Wang et al. have reported on co-catalytic systems composed *n*Bu_4_NI as the co-catalyst and pyridine-derivatives as the HBD that provided yields of up to 92% for reactions carried out over 12 h at 25 °C, 1.0 bars and using a 5:100 molar ratio of catalysts versus EP [61]. In our case, it is worth noting that figures similar to those described in previous works, e.g., yields of ca. 89%–94% and an excellent selectivity of ca. 98%, were obtained for reactions carried out over 20 h at 50 °C and 1.2 bars upon the use of DBU(1):BA(1) as the catalyst in a 10:100 molar ratio (see Entry 32 in Table 2).

**Figure 4:**
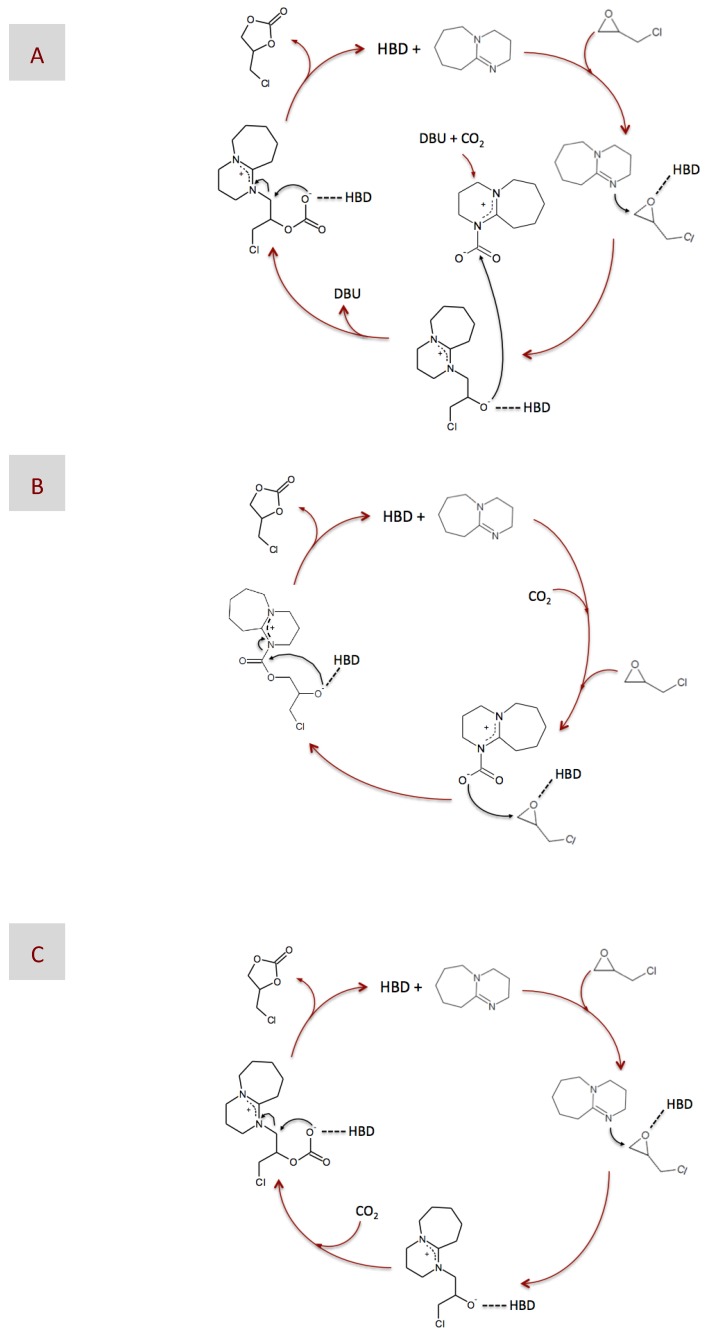
Plausible mechanisms of the chemical fixation of CO_2_ into epichlorohydrin (EP) catalyzed by DBU in the presence of a hydrogen bond donor (HBD), according to those proposed in previous works (**A**,**B**) and according to the NMR results obtained in this work (**C**).

After determining the catalytic performance of our DESs, we focused on analyzing the reaction mechanism. For this purpose, we used ^1^H and ^13^C NMR spectroscopy to study some of the reaction intermediates obtained from reactions catalyzed by either DBU or DBU-based DESs. In previous works, one of the more commonly-proposed mechanisms for reaction catalyzed by superbases consists of the formation of a ring-opened intermediate via nucleophilic attack, the nucleophiles being either regular ones like Br^−^, or the superbase itself (Figure 4A) [20,62,63], or a zwitterionic adduct formed between DBU and CO_2_ (Figure 4B) [20,61], on the less sterically hindered β -carbon atom of the epoxide ring. No matter what the nucleophile is, it is widely accepted that epoxide activation via H-bond interaction with an HBD favors the nucleophilic attack. Finally, carbonate formation occurs via either a second nucleophilic attack of the ring-opened intermediate to the zwitterionic adduct formed between DBU and CO_2_, i.e., in case the first nucleophilic attack was performed by regular nucleophiles (Figure 4A), or by molecular rearrangement, i.e., in case the first nucleophilic attack was performed by the zwitterionic DBU-CO_2_ adduct (Figure 4B). It is worth noting that the zwitterionic adduct formed between DBU and CO_2_ plays a significant role at some stage of the reaction in every one of the proposed mechanisms, i.e., as either the CO_2_ carrier in Figure 4A or the nucleophile in Figure 4B.

**Figure 5:**
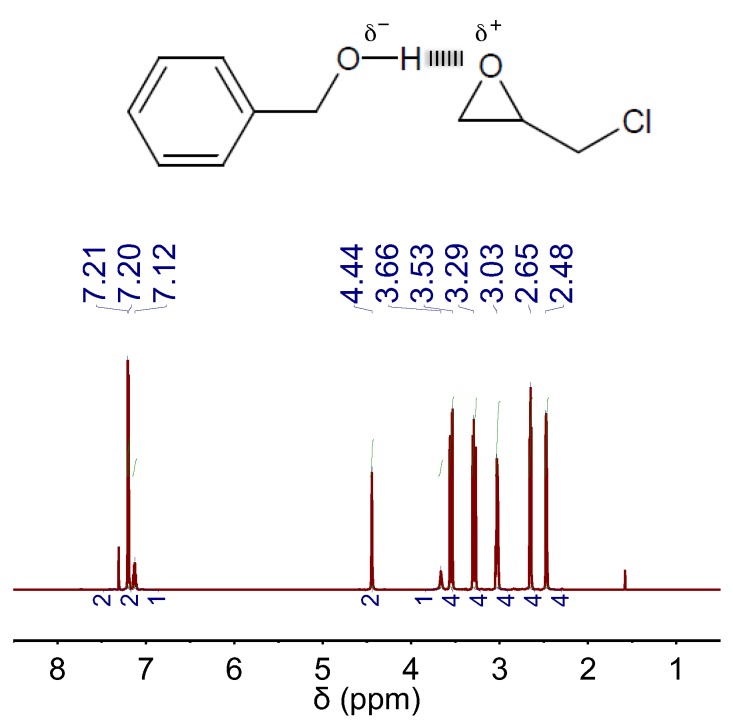
^1^H NMR spectra of a mixture of BA and EP in a molar ratio of 1:4. The chemical structures of EP and the H-bond complex formed between EP and BA are also included.

In our case, we first investigated the capability of BA to establish any kind of interaction with EP by ^1^H NMR spectroscopy. We found that, in a mixture of BA and EP, the peak positions of BA experienced some down-field chemical shift as compared to those of bare BA, whereas the peak positions of EP experienced some up-field chemical shift as compared to those of bare EP (compare Figure S1C with Figure 5). This occurrence was indicative of H-bond interactions between BA and EP, where BA plays the role of H-bond donor and EP plays the role of H-bond acceptor [21]. Actually, this situation resembled that occurring in DBU:BA DES where the well-known H-bond-donor- and H-bond-acceptor-role of, respectively, BA and DBU also resulted in similar shifts of their corresponding peaks (see Figure 1A).

With regard to the nucleophilic attack, none of the regular nucleophiles mentioned above were added to our reaction medium, so the candidates to assume that role were either the superbase itself or the zwitterionic DBU-CO_2_ adduct. In a previous work, ^13^C NMR spectroscopy allowed the identification of the adduct formation by the appearance of two peaks at 160.7 and 166.4 ppm corresponding to a carbamic and an amidinium carbon, respectively [64]. Unfortunately, XRD failed in adduct identification, obtaining instead the structure of the bicarbonate salt most likely as a consequence of adduct transformation into bicarbonate during crystallization in the presence of adventitious water. More recent ^13^C NMR studies assigned the peak at ca. 160 ppm to the bicarbonate anion, thus suggesting the preferred formation of [DBUH^+^][HCO_3_^−^] rather than of the zwitterionic DBU-CO_2_ adduct in both the presence and the absence of water [65]. In our case, attempts to identify the molecular structure of the zwitterionic DBU-CO_2_ adduct by ^13^C NMR spectroscopy also led to inconclusive results, i.e., we found a peak at ca. 167 ppm corresponding to an amidinium carbon, but none at ca. 160 ppm, thus disregarding the presence of the carbamic carbon and of any bicarbonate salt. It is also worth noting that the peak at ca. 167 ppm appeared as soon as DBU, in either its bare or DES-form, was mixed with EP, i.e., this is, not only in the reaction conditions, but also before the addition of CO_2_, further supporting the unlikely occurrence of both the zwitterionic DBU-CO_2_ adduct and the bicarbonate salt in our case. 

**Figure 6:**
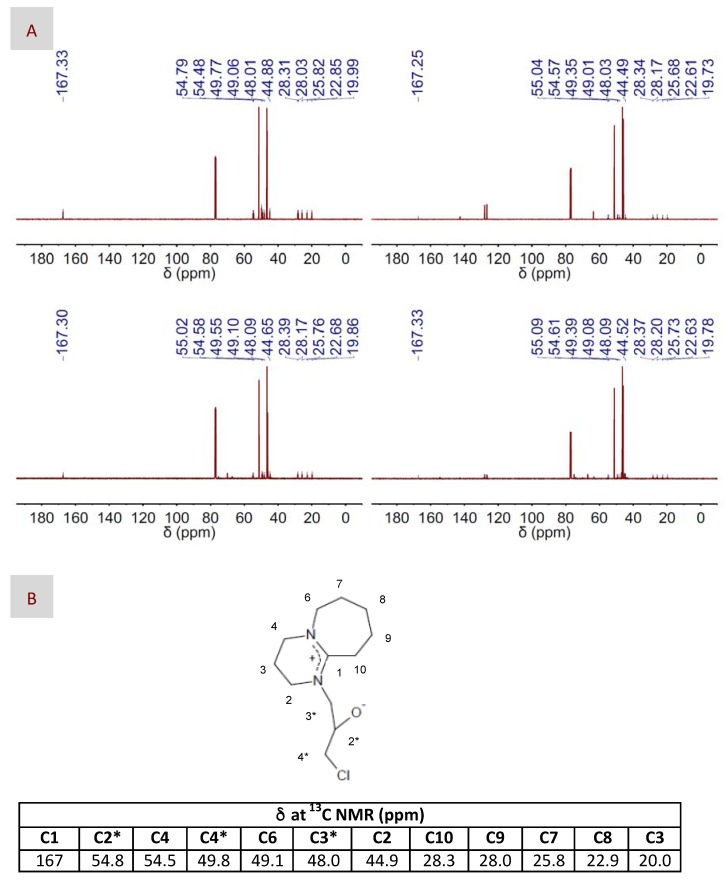
(**A**) ^13^C NMR spectra of the intermediate observed in the reaction mixture composed of EP and DBU, in either its bare (left column) or DES-form (right column), and in the absence (top panel) or the presence (bottom panel) of CO_2_; (**B**) The chemical structure proposed for the intermediate, as well as the chemical shifts assigned to the structure in the ^13^C NMR spectra are also included for better visualization of the corresponding peaks at the NMR spectra.

Assuming the absence of the zwitterionic DBU-CO_2_ adduct, one could disregard the mechanisms described in Figure 4A,B, i.e., where the zwitterionic DBU-CO_2_ adduct acted as either the CO_2_ carrier or the nucleophile, respectively, as the more plausible ones in our case. Moreover, our only choice to play the role of nucleophile was the superbase itself. Actually, most of the carbons of the intermediate resulting from this nucleophilic attack could be assigned to the peaks appearing at the ^13^C NMR spectra depicted in Figure 6. Interestingly, we observed the formation of this intermediate for DBU in both its bare and DES-based form, thus revealing why, in our case, epoxide activation via H-bond interaction with BA provides a negligible advantage, i.e., in terms of reaction yield, as compared to non-activated systems. Based on the above-discussed NMR results, the mechanism proposed in this work is that depicted in Figure 4C, where DBU was responsible for the nucleophilic attack to EP, whether HBD-activated or not, and CO_2_ was fixed into the resulting intermediate without the aid of any eventual carrier. At this stage, a question that may arise is why the formation of the zwitterionic DBU-CO_2_ adduct proposed in every previous mechanism was missing in our case. We hypothesized that the prompt formation of the intermediate, i.e., detected at the very early stages of the reaction, even before CO_2_ addition, made DBU no longer available in the reaction medium for interacting with CO_2_ and form the zwitterionic DBU-CO_2_ adduct. This mechanism was in agreement with those proposed for catalysts like imidazolium halide and silanediol [66,67]. 

**Figure 7:**
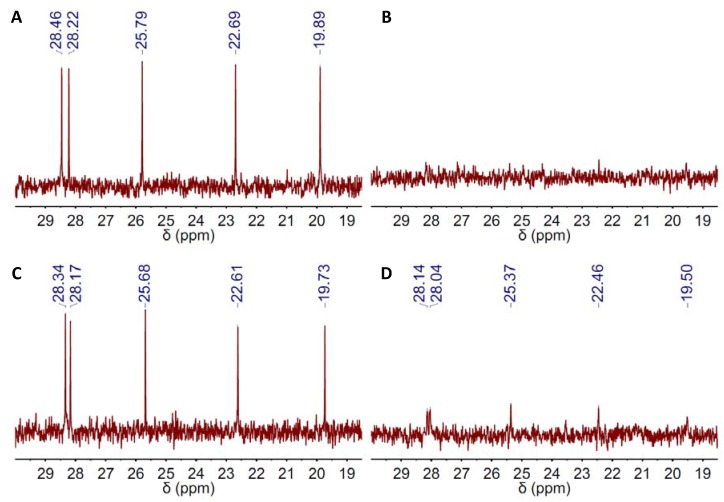
^13^C NMR spectra of the reaction mixture before and after submission of the reaction mixture to 100 °C over 2 h in a 1.2-bar CO_2_ atmosphere using either (**A**,**B**) DBU or (**C**,**D**) DBU(1):BA(1) DES as catalysts. The region of chemical shifts displayed in the spectra just ranged from ca. 20–30 ppm because neither EP, nor BA exhibit peaks at that region.

Finally and with regard to whether the catalytic performance of DBU-based DESs improved as compared to that of bare DBU, Table 2 shows that the improvement indeed occurred, but only in a small percentage. Under these circumstances, the question may arise as to whether DBU-based DESs really offered any further advantage as compared to bare DBU. In this regard, one may notice that different works have expressed serious concerns about the thermal stability of superbases like DBU in certain solvents. In our case, we studied the thermal stability of DBU by ^13^C NMR spectroscopy in a region of chemical shifts, e.g., ca. 20–30 ppm, that provides an excellent fingerprint of DBU because neither EP, nor any of the HBDs exhibit peaks at that region. Thus, both bare and DES-based DBU exhibited four peaks within the 20–30-ppm range of the ^13^C NMR spectra (Figure S1A, Figure 1A), which became five after mixing with EP (Figure 7). DBU stability assessment was done by comparing the intensity of these five peaks before and after submission of the reaction mixture to 100 °C over 2 h in CO_2_ atmosphere. Interestingly, these five peaks fully disappeared in the bare-DBU case, whereas they remained in the DES one (Figure 7). Nonetheless, it is worth noting the low intensity of these five peaks in this latter case, as well, so one should yet question the DBU stability even in its DES form.

## 3. Materials and Methods

### 3.1. Materials

BA, EG, MDEA, TBD, DBU and EP were all supplied by Sigma-Aldrich (St. Luis, AZ, USA) and used as received. CO_2_ with a purity of 99.9999% was from Air Liquide.

### 3.2. Preparation of DESs

DESs were prepared upon the dropwise addition of the HBD onto the superbase in an ice bath to avoid acid-base/exothermic reactions. Different DESs were prepared by mixing the HBD and the superbase of choice in different molar ratios. Afterwards, the mixtures were stirred over 5 min until complete formation of homogeneous and transparent liquid solutions.

### 3.3. CO_2_ Absorption on DESs

CO_2_ absorption on DESs was performed placing a certain amount of DESs in a closed reactor and flowing CO_2_ into the reactor at 0.2 L/min and at room temperature. The CO_2_ flow was maintained until we observed a negligible gain of weight. The amount of absorbed CO_2_ on to every DES was obtained by ^1^H NMR spectroscopy.

### 3.4. CO_2_ Fixation to EP

All of the cycloaddition reactions were carried out in a Büchi Glas Uster high-pressure reactor with a 25-mL glass vessel and a cyclone 075 stirring unit. In a typical synthesis, the reactor was first fed with EP and the superbase, in either its bare or DES form, in the desired catalyst molar ratio, 1:100 or 10:100, and then purged with CO_2_. Afterwards, the autoclave was heated to the working temperatures, 50 or 100 °C, and the working pressure, 1.2 or 6 bars, was reached upon further CO_2_ introduction. The reaction was carried out over 1, 2 or 20 h depending on the reaction conditions of choice. Once the reaction ended, the temperature was cooled to 20 °C, and the remaining CO_2_ was slowly released. The analysis of the products resulting after reaction, as well as the final conversion values were obtained by ^1^H NMR spectroscopy and GC/Ms of the crude medium.

### 3.5. Characterization

^1^H and ^13^C NMR spectra were recorded using a Bruker spectrometer DR X-500 (Bruker, Billerica, MA, USA). For this purpose, the liquid molecules used for DES, the resulting DESs and some of the DESs loaded with CO_2_, e.g., DBU(1):BA(1), DBU(1):EG(1) and TBD(1):BA(1), were placed in a capillary tube using CDCl_3_ as the external reference. CDCl_3_ was used as not only the solvent, but also an internal reference, so the deuterium signal was used for locking and shimming the sample, in those cases where the samples exhibited high viscosity, e.g., all of the remaining DESs loaded with CO_2_. GC analyses were performed on a Varian 3800 gas chromatograph/1200L triple quadrupole mass spectrometer (Agilent, Santa Clara, CA, USA, equipped with a split/splitless injector). The data system contains all of the software required for calibration, collection of GC/MS spectra and data processing for qualitative and quantitative analysis. The chromatographic separation was performed by a Factor IV capillary column (30 m × 0.25 mm i.d., 0.25-μm film thickness). Helium was used as the carrier gas at a flow rate of 1.0 mL/min. Argon was used as the collision gas. The injector temperature was set at 250 °C, and 1 μL was injected in undividable mode for 1 min. The oven temperature program used for analysis was the following; initial temperature set to 40 °C, hold at 40 °C for 2.1 min, heated to 250 °C with a ramp of 25 °C/min and hold at 250 °C for 6 min.

## 4. Conclusions

In this work, DESs composed of superbases, e.g., TBD or DBU, and alcohols, e.g., BA, EG or MDEA, showed a high performance as CO_2_ sorbents. The performance of some of these DESs as catalysts in the chemical fixation of CO_2_ to EP was also evaluated. DESs proved highly effective as catalysts, so that the resulting carbonate was obtained with yields above 90% and selectivities approaching 100% after only two hours of reaction in pseudo-mild reaction conditions, e.g., 1.2 bars and 100 °C, and after 20 hours if the reaction conditions of choice were even milder, e.g., 1.2 bars and 50 °C. The study of the reactions catalyzed by DBU, either in its bare form or forming a DES with BA, allowed the identification of the intermediate resulting from the nucleophilic attack of DBU to EP. In our case, we were not able to identify the zwitterionic DBU-CO_2_ adduct, the presence of which was quite commonly proposed as either the nucleophile or CO_2_ carrier. We hypothesized that the prompt formation of the intermediate, i.e., detected at the very early stages of the reaction, even before CO_2_ addition, made DBU no longer available in the reaction medium for interacting with CO_2_ and thus forms the zwitterionic DBU-CO_2_ adduct. Based on these results, we proposed an alternative mechanism where the role played by the zwitterionic DBU-CO_2_ adduct was neither as nucleophile, nor as CO_2_ carrier. Finally, the investigation of the catalyst after reaction revealed that the stability of DBU in its bare form was quite poor, whereas in DES form, it experienced a certain improvement, not enough yet to avoid questioning its overall capability as a catalyst.

## Figures and Tables

**Table 1:** CO_2_ absorption capacity of the different DBU- and TBD-based DESs as calculated using data coming from the integral of ^1^H NMR spectra of DESs after CO2 loading. EG, ethylene glycol; MDEA, methyldiethanolamine.

DES	mol CO_2_/mol Superbase
DBU(1):BA(1)	0.28–0.25
DBU(1):BA(4)	0.90–1
DBU(1):EG(1)	0.33–0.3
DBU(1):EG(4)	0.98–1
DBU(1):MDEA(2)	0.50–0.5
TBD(1):BA(1)	0.45–0.5
TBD(1):BA(4)	0.96–1
TBD(1):EG(1)	0.50–0.5
TBD(1):EG(4)	1–1
TBD(1):MDEA(2)	0.52–0.5

**Table 2:** Yield and selectivity found for the chemical fixation of CO_2_ into EP using DBU and TBD, in either their bare form or in the form of DES, as catalysts and different experimental conditions.

Entry	Type of Catalyst	Molar Ratio of Catalyst Versus Reagent	T (°C)	t (h)	P (bars)	Yield (%)	Selectivity (%)
#1	DBU	1:100	100	1	6	60 ^a^/64 ^b^	98 ^a^/98 ^b^
#2	DBU	1:100	100	2	6	82 ^a^/88 ^b^	98 ^a^/98 ^b^
#3	DBU	1:100	100	1	1.2	72 ^a^/81 ^b^	99 ^a^/99 ^b^
#4	DBU	1:100	100	2	1.2	84 ^a^/87 ^b^	99 ^a^/99 ^b^
#5	DBU	1:100	50	2	1.2	27 ^a^/9 ^b^	90 ^a^/90 ^b^
#6	DBU	1:100	50	20	1.2	49 ^a^/52 ^b^	97 ^a^/97 ^b^
#7	DBU	10:100	50	2	1.2	42 ^a^/43 ^b^	75 ^a^/75 ^b^
#8	DBU	10:100	50	20	1.2	92 ^a^/95 ^b^	98 ^a^/98 ^b^
#9	TBD	1:100	100	2	1.2	93 ^a^/88 ^b^	99 ^a^/99 ^b^
#10	TBD	1:100	50	2	1.2	8 ^a^/10 ^b^	88 ^a^/88 ^b^
#11	TBD	1:100	50	20	1.2	50 ^a^/58 ^b^	99 ^a^/99 ^b^
#12	TBD	10:100	50	2	1.2	35 ^a^/41 ^b^	86 ^a^/86 ^b^
#13	TBD	10:100	50	20	1.2	97 ^a^/99 ^b^	97 ^a^/97 ^b^
#14	DBU(1):BA(1)	1:100	100	1	6	67 ^a^/62 ^b^	98 ^a^/98 ^b^
#15	DBU(1):BA(1)	1:100	100	2	6	83 ^a^/85 ^b^	99 ^a^/99 ^b^
#16	DBU(1):BA(1)	1:100	100	1	1.2	73 ^a^/70 ^b^	98 ^a^/98 ^b^
#17	DBU(1):BA(1)	1:100	100	2	1.2	86 ^a^/93 ^b^	99 ^a^/99 ^b^
#18	DBU(1):EG(1)	1:100	100	1	6	67 ^a^/74 ^b^	98 ^a^/98 ^b^
#19	DBU(1):EG(1)	1:100	100	2	6	84 ^a^/86 ^b^	99 ^a^/99 ^b^
#20	DBU(1):EG(1)	1:100	100	1	1.2	73 ^a^/66 ^b^	98 ^a^/98 ^b^
#21	DBU(1):EG(1)	1:100	100	2	1.2	83 ^a^/86 ^b^	98 ^a^/98 ^b^
#22	DBU(1):MDEA(2)	1:100	100	1	6	79 ^a^/79 ^b^	97 ^a^/97 ^b^
#23	DBU(1):MDEA(2)	1:100	100	2	6	88 ^a^/90 ^b^	97 ^a^/97 ^b^
#24	DBU(1):MDEA(2)	1:100	100	1	1.2	82 ^a^/81 ^b^	96 ^a^/96 ^b^
#25	DBU(1):MDEA(2)	1:100	100	2	1.2	91 ^a^/93 ^b^	97 ^a^/97 ^b^
#26	TBD(1):BA(1)	1:100	100	2	1.2	98 ^a^/96 ^b^	98 ^a^/98 ^b^
#27	TBD(1):EG(1)	1:100	100	2	1.2	94 ^a^/87 ^b^	98 ^a^/98 ^b^
#28	TBD(1):MDEA(2)	1:100	100	2	1.2	90 ^a^/97 ^b^	97 ^a^/97 ^b^
#29	DBU(1):BA(1)	1:100	50	2	1.2	11 ^a^/12 ^b^	87 ^a^/87 ^b^
#30	DBU(1):BA(1)	1:100	50	20	1.2	58 ^a^/62 ^b^	98 ^a^/98 ^b^
#31	DBU(1):BA(1)	10:100	50	2	1.2	57 ^a^/57 ^b^	96 ^a^/96 ^b^
#32	DBU(1):BA(1)	10:100	50	20	1.2	89 ^a^/94 ^b^	98 ^a^/98 ^b^
#33	TBD(1):BA(1)	1:100	50	2	1.2	8 ^a^/6 ^b^	88 ^a^/88 ^b^
#34	TBD(1):BA(1)	1:100	50	20	1.2	44 ^a^/46 ^b^	97 ^a^/97 ^b^
#35	TBD(1):BA(1)	10:100	50	2	1.2	6 ^a^/21 ^b^	76 ^a^/76 ^b^
#36	TBD(1):BA(1)	10:100	50	20	1.2	93 ^a^/86 ^b^	93 ^a^/93 ^b^

^a^ As obtained from NMR spectroscopy ^b^ As obtained from GC.

## Supplementary Materials

The following are available online at www.mdpi.com/1996-1944/10/7/759/s1. Figure S1: Chemical structures of molecules used for preparation of DESs, e.g., DBU and TBD as the ammonium salts and ethylene glycol (EG), benzyl alcohol (BA) and methyldiethanolamine (MDEA) as the HBDs. The chemical structure of epichlorohydrin (EP) was also included. Figure S2: ^1^H and ^13^C NMR spectra (left and right columns, respectively) of DBU(1):BA(4) before (A,C) and after (B,D) CO_2_ absorption. Figure S3: ^1^H and ^13^C NMR spectra (left and right columns, respectively) of DBU(1):EG(1) before (A,C) and after (B,D) CO_2_ absorption. Figure S4: ^1^H and ^13^C NMR spectra (left and right columns, respectively) of DBU(1):EG(4) before (A,C) and after (B,D) CO_2_ absorption. Figure S5: ^1^H and ^13^C NMR spectra (left and right columns, respectively) of DBU(1):MDEA(2) before (A,C) and after (B,D) CO_2_ absorption. Figure S6: ^1^H and ^13^C NMR spectra (left and right columns, respectively) of TBD(1):BA(4) before (A,C) and after (B,D) CO_2_ absorption. Figure S7: ^1^H and ^13^C NMR spectra (left and right columns, respectively) of TBD(1):EG(1) before (A,C) and after (B,D) CO_2_ absorption. Figure S8: ^1^H and ^13^C NMR spectra (left and right columns, respectively) of TBD(1):EG(4) before (A,C) and after (B,D) CO_2_ absorption. Figure S9: ^1^H and ^13^C NMR spectra (left and right columns, respectively) of TBD(1):MDEA(2) before (A,C) and after (B,D) CO_2_ absorption. Figure S10: Scheme representing the chemical structures of (left) the DES resulting from H-bond interaction between a superbase B, e.g., DBU or TBD, and an HBD, e.g., EG or MDEA, and the salts resulting after CO_2_ absorption. Table S1: Chemical shifts obtained from the ^1^H NMR spectra of the individual components, e.g., DBU and BA, the DESs resulting after their mixture in 1:1 and 1:4 molar ratios, e.g., DBU(1):BA(1) and DBU(1):BA(4), and the same DESs after loading with CO_2_. Table S2: Chemical shifts obtained from the ^1^H NMR spectra of the individual components, e.g., TBD and BA, the DESs resulting after their mixture in 1:1 and 1:4 molar ratios, e.g., TBD(1):BA(1) and TBD(1):BA(4), and the same DESs after loading with CO_2_. Table S3: Chemical shifts obtained from the ^1^H NMR spectra of the individual components, e.g., DBU and EG, the DESs resulting after their mixture in 1:1 and 1:4 molar ratios, e.g., DBU(1):EG(1) and DBU(1):EG(4), and the same DESs after loading with CO_2_. Table S4: Chemical shifts obtained from the ^1^H NMR spectra of the individual components, e.g., DBU and NMDEA, the DES resulting after their mixture in a 1:2 molar ratio, e.g., DBU(1): MDEA(2), and the same DES after loading with CO_2_. Table S5: Chemical shifts obtained from the ^1^H NMR spectra of the individual components, e.g., TBD and EG, the DESs resulting after their mixture in 1:1 and 1:4 molar ratios, e.g., TBD(1):EG(1) and TBD(1):EG(4), and the same DESs after loading with CO_2_. Table S6: Chemical shifts obtained from the ^1^H NMR spectra of the individual components, e.g., TBD and MDEA, the DESs resulting after their mixture in a 1:2 molar ratio, e.g., TBD(1):MDEA(2), and the same DES after loading with CO_2_. Table S7: Chemical shifts obtained from the ^13^C NMR spectra of the individual components, e.g., DBU and BA, the DESs resulting after their mixture in 1:1 and 1:4 molar ratios, e.g., DBU(1):BA(1) and DBU(1):BA(4), and the same DESs after loading with CO_2_. Table S8: Chemical shifts obtained from the ^13^C NMR spectra of the individual components, e.g., TBD and BA, the DESs resulting after their mixture in 1:1 and 1:4 molar ratios, e.g., TBD(1):BA(1) and TBD(1):BA(4), and the same DESs after loading with CO_2_. Table S9: Chemical shifts obtained from the ^13^C NMR spectra of the individual components, e.g., DBU and EG, the DESs resulting after their mixture in 1:1 and 1:4 molar ratios, e.g., DBU(1):EG(1) and DBU(1):EG(4), and the same DESs after loading with CO_2_. Table S10: Chemical shifts obtained from the ^13^C NMR spectra of the individual components, e.g., DBU and NMDEA, the DES resulting after their mixture in a 1:2 molar ratio, e.g., DBU(1):MDEA(2), and the same DES after loading with CO_2_. Table S11: Chemical shifts obtained from the ^13^C NMR spectra of the individual components, e.g., TBD and EG, the DESs resulting after their mixture in 1:1 and 1:4 molar ratios, e.g., TBD(1):EG(1) and TBD(1):EG(4), and the same DESs after loading with CO_2_. Table S12: Chemical shifts obtained from the ^13^C NMR spectra of the individual components, e.g., TBD and NMDEA, the DES resulting after their mixture in a 1:2 molar ratio, e.g., TBD(1):MDEA(2), and the same DES after loading with CO_2_.
