# Supplementary material for: Study of Superbase-Based Deep Eutectic Solvents as the Catalyst in the Chemical Fixation of CO2 into Cyclic Carbonates under Mild Conditions
Source: Materials (Basel). 2017 Jul 7;10(7):759. doi: 10.3390/ma10070759 (PMC5551802; doi:10.3390/ma10070759)
Supplement: Supplementary file 1 [file materials-10-00759-s001.pdf]

Figure S1: Chemical structures of molecules used for preparation of DESs – e.g. DBU and TBD as the ammonium salts, and ethylene glycol (EG), benzyl alcohol (BA), and methyldiethanolamine (MDEA) as the HBDs. The chemical structure of epichlorohydrin (EP) was also included.

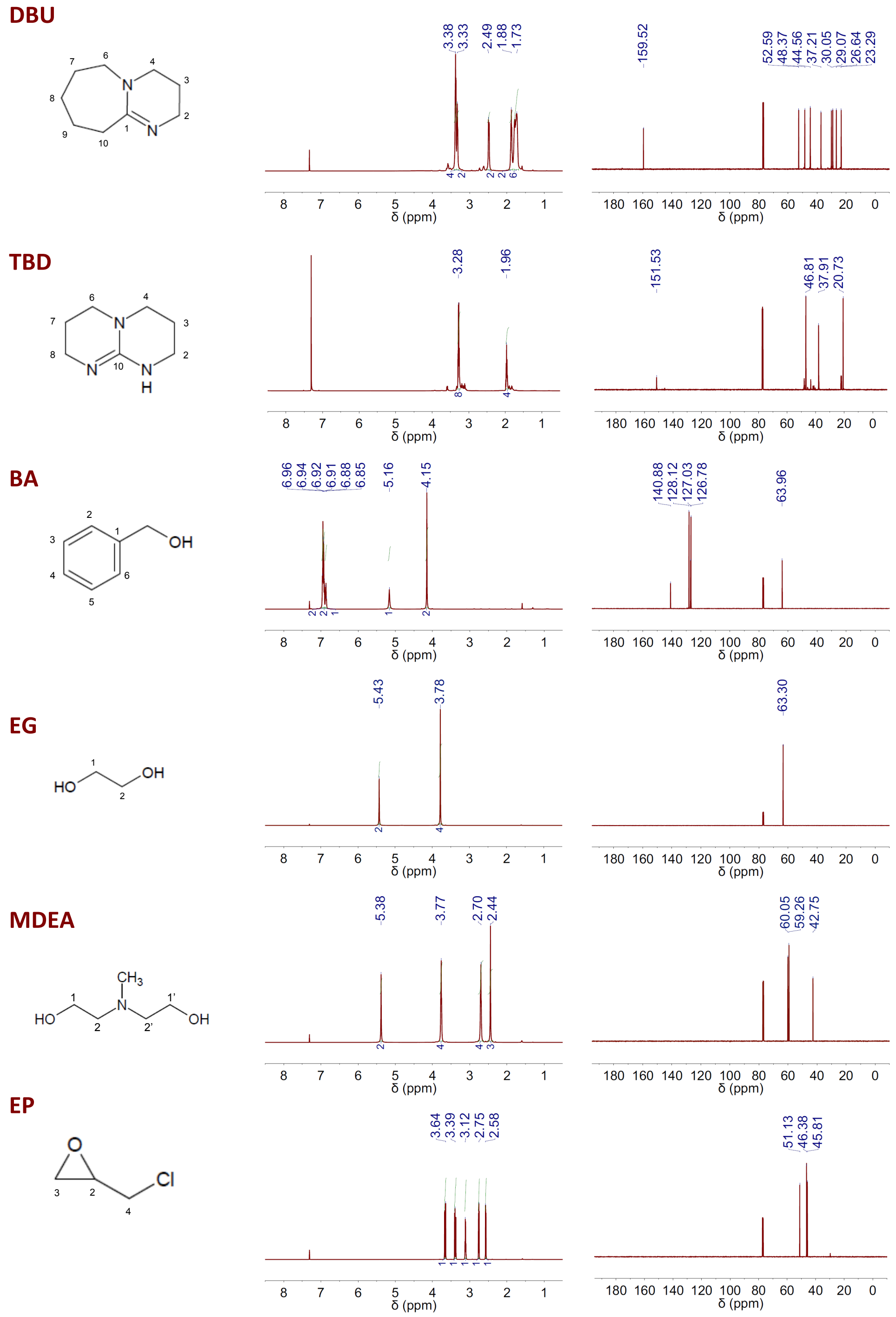

Figure S2: ^{1}H and ^{13}C NMR spectra (left and right columns, respectively) of DBU(1):BA(4) before (A, C) and after (B, D) CO_2 absorption.

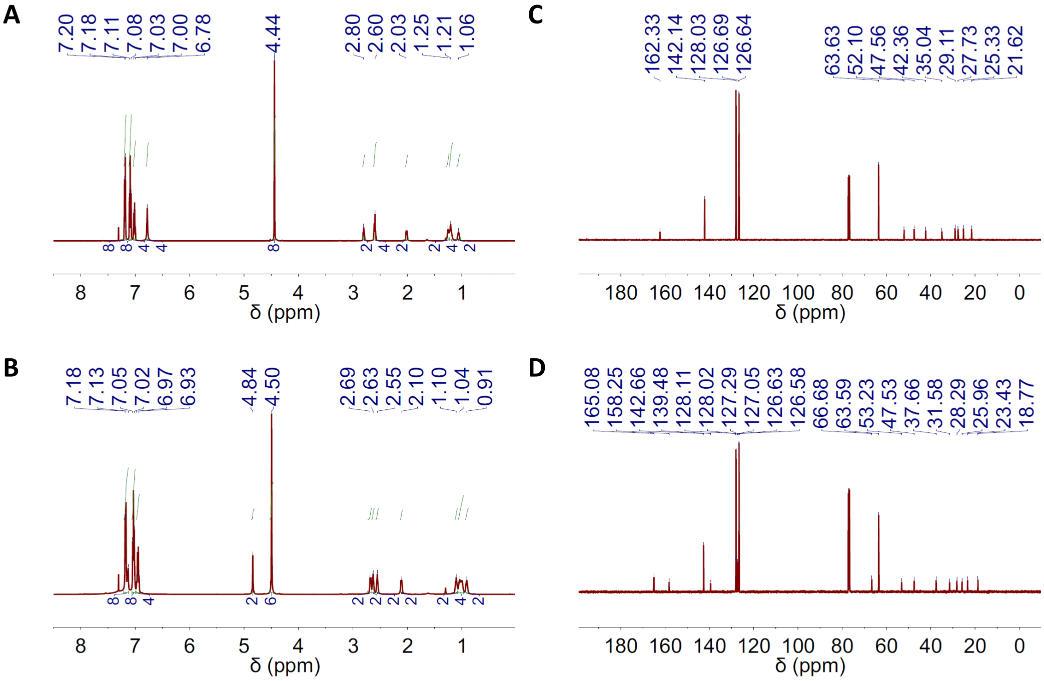

Figure S3: ^{1}H and ^{13}C NMR spectra (left and right columns, respectively) of DBU(1):EG(1) before (A, C) and after (B, D) CO_2 absorption.

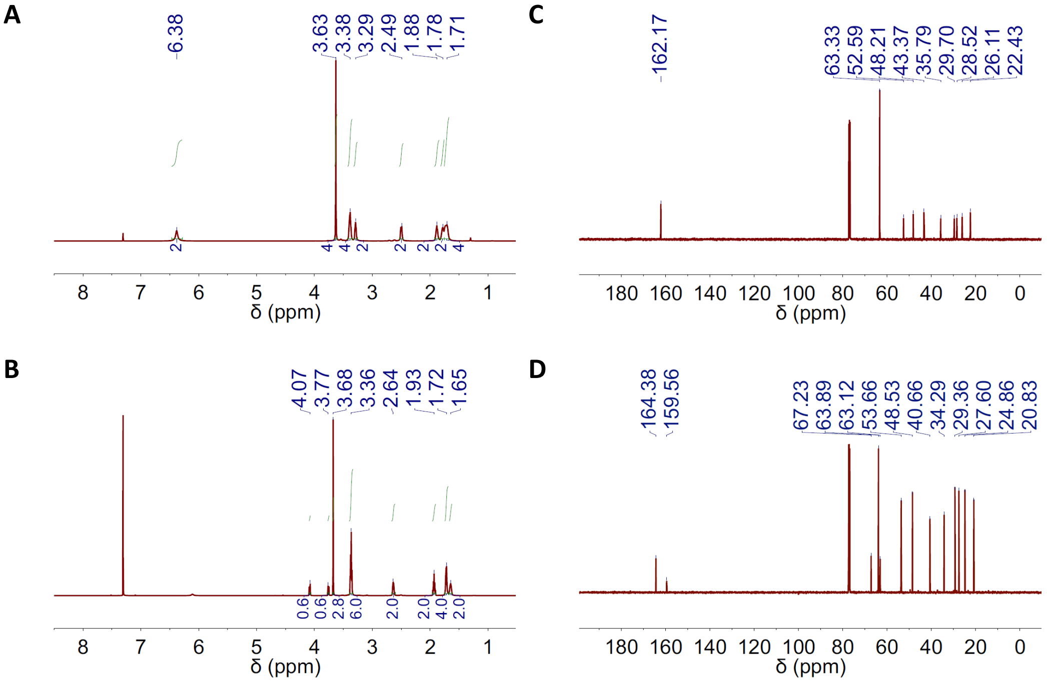

Figure S4: ^{1}H and ^{13}C NMR spectra (left and right columns, respectively) of DBU(1):EG(4) before (A, C) and after (B, D) CO_2 absorption.

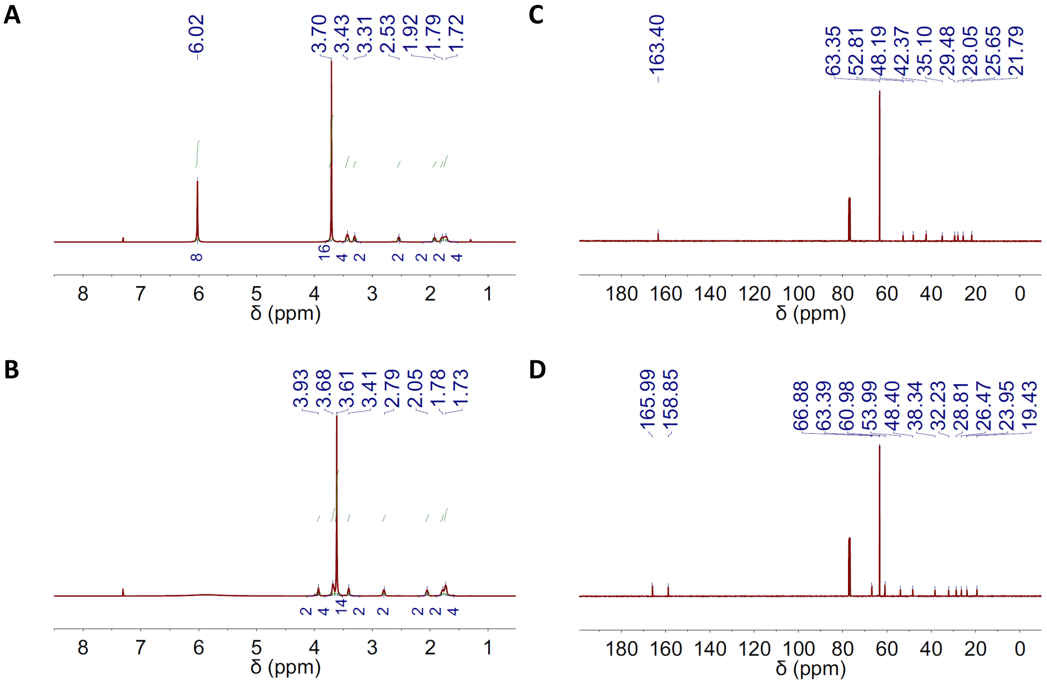

Figure S5: ^{1}H and ^{13}C NMR spectra (left and right columns, respectively) of DBU(1):MDEA(2) before (A, C) and after (B, D) CO_2 absorption.

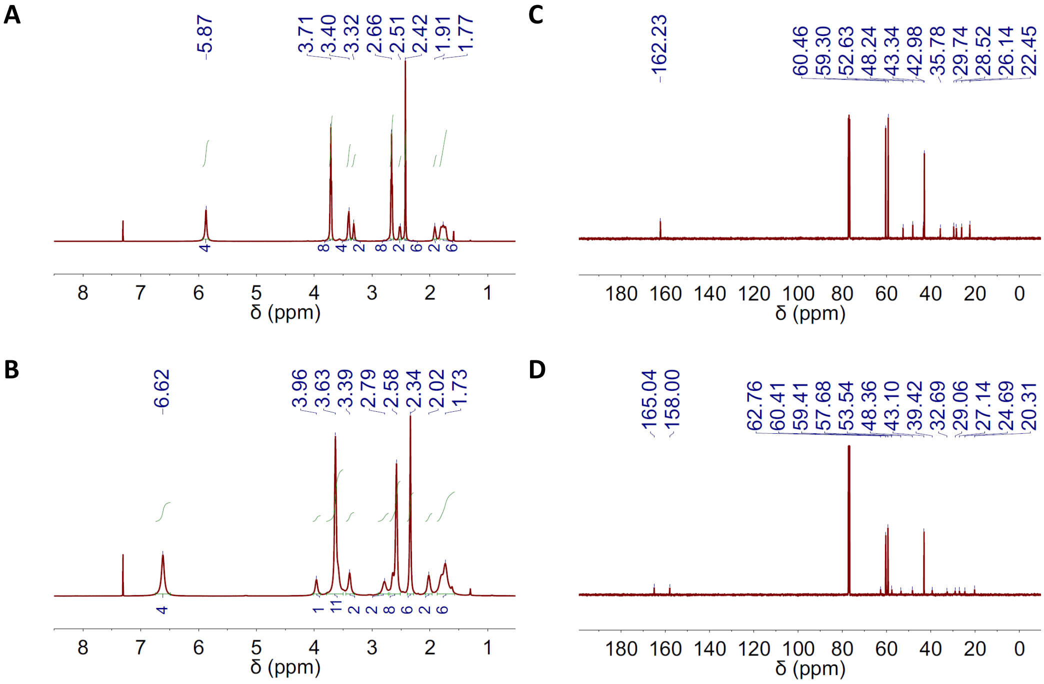

Figure S6: ^{1}H and ^{13}C NMR spectra (left and right columns, respectively) of TBD(1):BA(4) before (A, C) and after (B, D) CO_2 absorption.

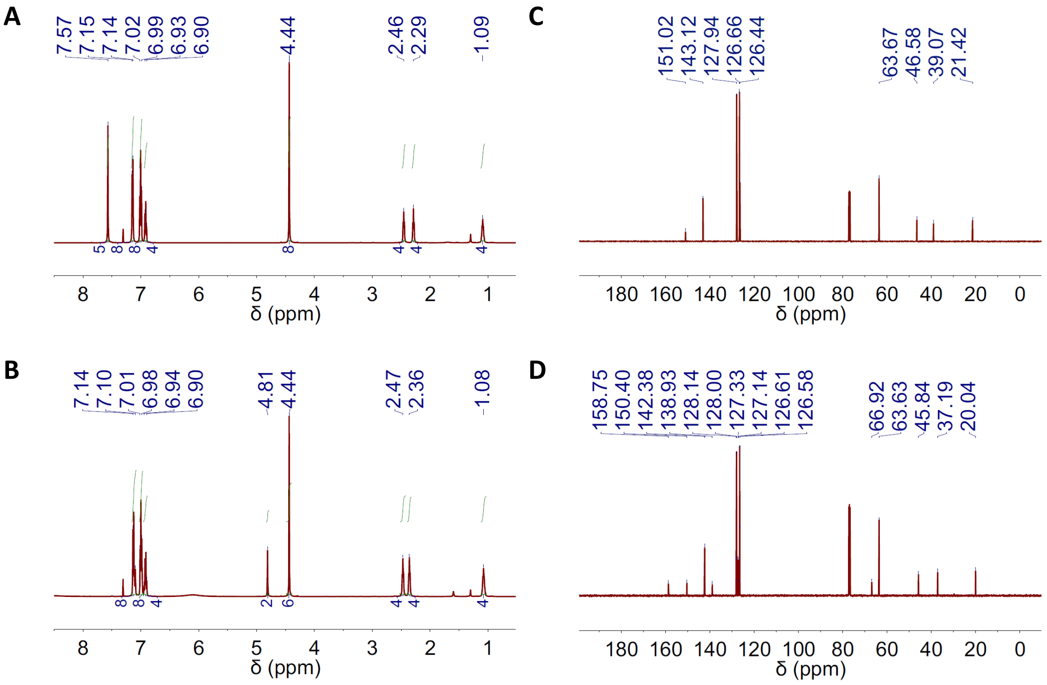

Figure S7: ^{1}H and ^{13}C NMR spectra (left and right columns, respectively) of TBD(1):EG(1) before (A, C) and after (B, D) CO_2 absorption.

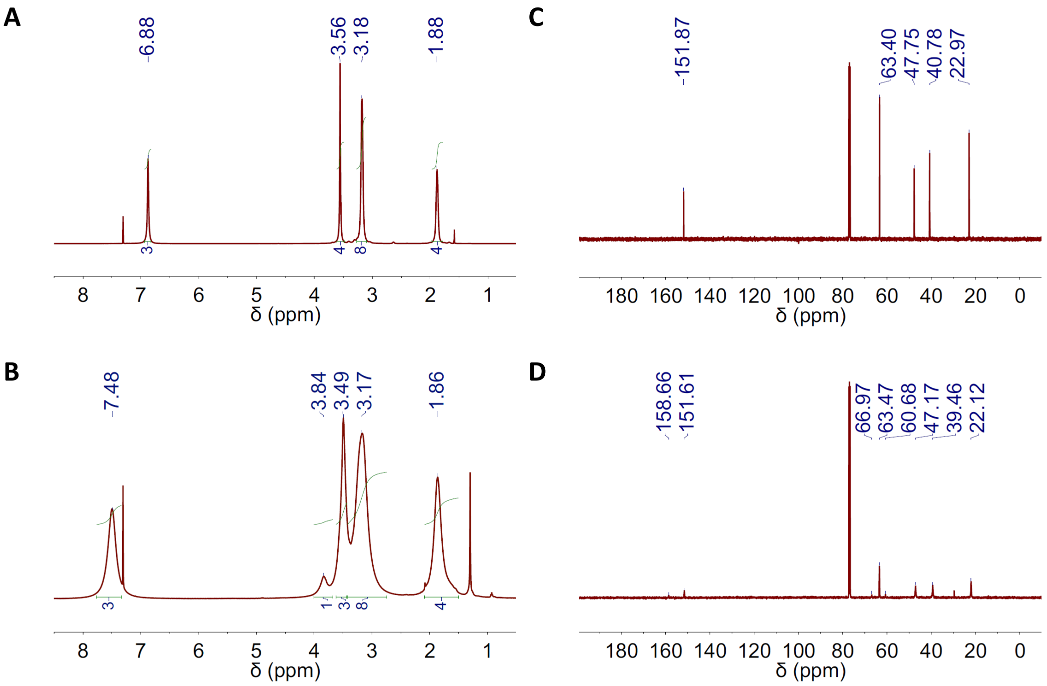

Figure S8: ^{1}H and ^{13}C NMR spectra (left and right columns, respectively) of TBD(1):EG(4) before (A, C) and after (B, D) CO_2 absorption.

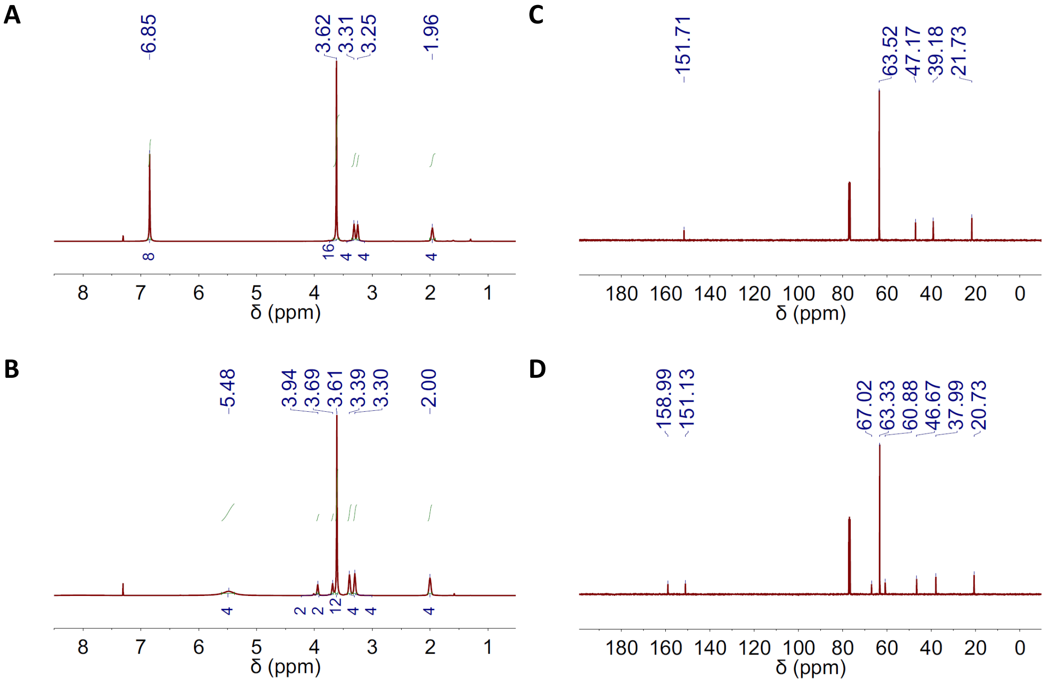

Figure S9: ^{1}H and ^{13}C NMR spectra (left and right columns, respectively) of TBD(1):MDEA(2) before (A, C) and after (B, D) CO_2 absorption.

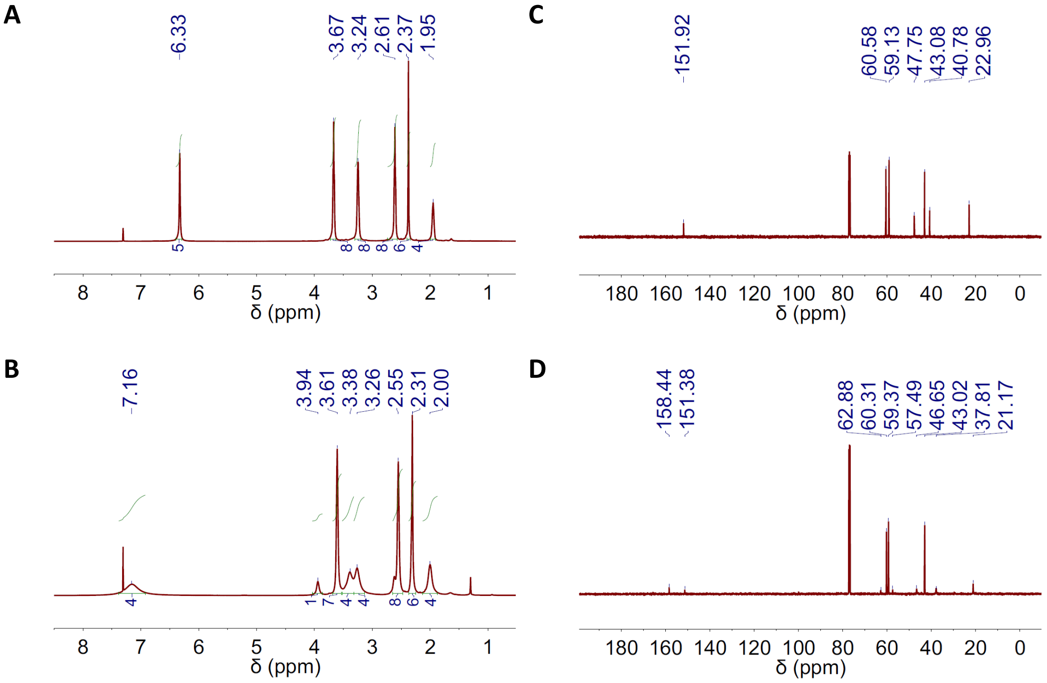

Figure S10: Scheme representing the chemical structures of (left) the DES resulting from H-bond interaction between a superbase B – e.g. DBU or TBD – and an HBD – e.g. EG or MDEA, and the salts resulting after CO_2 absorption.

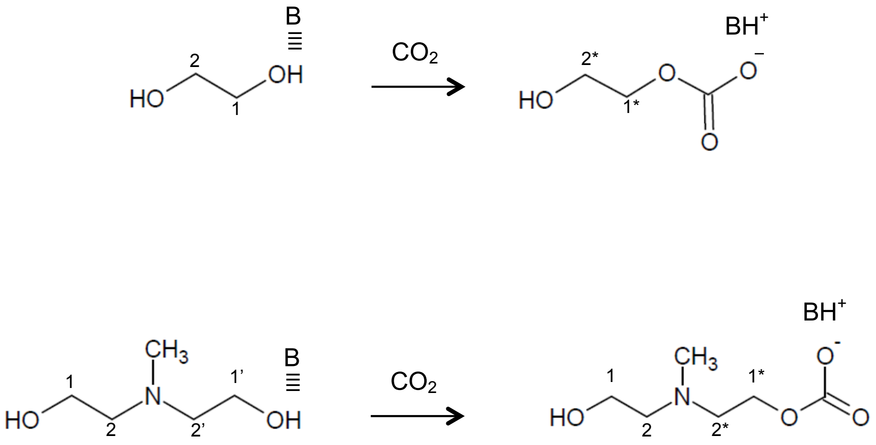

Table S1: Chemical shifts obtained from the ^{1}H NMR spectra of the individual components – e.g. DBU and BA, the DESs resulting after their mixture in 1:1 and 1:4 molar ratios – e.g. DBU(1):BA(1) and DBU(1):BA(4), and the same DESs after loading with CO_2.

Sample	2,3,4,6,7,8,9,10-octahydropyrimido[1,2-*a*]azepine (DBU)								Benzyl alcohol (BA)					
	**H** at C2 (CH_2)	**H** at C4 (CH_2)	**H** at C6 (CH_2)	**H** at C10 (CH_2)	**H** at C3 (CH_2)	**H** at C9 (CH_2)	**H** at C8 (CH_2)	**H** at C7 (CH_2)	**H** at C3&C5 2×(CH)	**H** at C4 (CH)	**H** at C2&C6 2×(CH)	**H** at CH_2-OH	**H** at OH	**H** at CH_2-OCOO-
**DBU**	3.38 (4H)		3.33 (2H)	2.49 (2H)	1.88 (2H)	1.73 (6H)								
**BA**									6.96-6.94 (2H)	6.88-6.85 (1H)	6.92-6.91 (2H)	4.14 (2H)	5.20 (1H)	
**DBU(1):BA(1)**	3.07 (2H)	2.97 (4H)		2.28 (2H)	1.56 (2H)	1.47 (4H)		1.36 (2H)	7.39-7.37 (2H)	7.16-7.13 (1H)	7.26-7.23 (2H)	4.63 (2H)	7.62 (1H)	-
**DBU(1):BA(1) + CO_2**	3.30 (6H)			2.59 (2H)	1.88 (2H)	1.69 (4H)		1.61 (2H)	7.42-7.38 (2H)	7.26-7.22 (1H)	7.34-7.28 (2H)	4.68 (1.5H)	**-**	5.03 (0.5H)
**DBU(1):BA(4)**	2.80 (2H)	2.60 (4H)		2.03 (2H)	1.25 (2H)	1.21 (4H)		1.06 (2H)	7.20-7.18 (8H)	7.03-7 (4H)	7.11-7.08 (8H)	4.44 (8H)	6.78 (4H)	-
**DBU(1):BA(4) + CO_2**	2.69 (2H)	2.63 (2H)	2.55 (2H)	2.10 (2H)	1.10 (2H)	1.04 (4H)		0.91 (2H)	7.18-7.13 (8H)	6.97-6.93 (4H)	7.05-7.02 (8H)	4.50 (6H)	-	4.86 (2H)

Table S2: Chemical shifts obtained from the ^{1}H NMR spectra of the individual components – e.g. TBD and BA, the DESs resulting after their mixture in 1:1 and 1:4 molar ratios – e.g. TBD(1):BA(1) and TBD(1):BA(4), and the same DESs after loading with CO_2.

Sample	2,3,4,6,7,8-Hexahydro-1H-pyrimido[1,2-a]pyrimidine (TBD)			Benzyl alcohol (BA)					
	**H** at C2&C8 (CH_2)	**H** at C4&C6 (CH_2)	**H** at C3&C5 (CH_2)	**H** at C3&C5 2×(CH)	**H** at C4 (CH)	**H** at C2&C6 2×(CH)	**H** at CH_2-OH	**H** at OH	**H** at -CH_2-OCOO-
**TBD**	3.28 (8H)		1.96 (4H)	-	-	-	-	-	-
**BA**	-	-	-	6.96-6.94 (2H)	6.88-6.85 (1H)	6.92-6.91 (2H)	4.14 (2H)	5.20 (1H)	-
**TBD(1):BA(1)**	2.92 (4H)	2.77 (4H)	1.55 (4H)	7.32-7.31 (2H)	7.09-7.06 (1H)	7.19-7.16 (2H)	4.59 (2H)	6.93 (2H)[a]	-
**TBD(1):BA(1) + CO2**	3.27 (8H)		1.98 (4H)	7.44-7.39 (2H)	7.29-7.22 (1H)	7.36-7.32 (2H)	4.71 (1H)	-	5.05 (1H)
**TBD(1):BA(4)**	2.46 (4H)	2.29 (4H)	1.09 (4H)	7.15-7.14 (8H)	6.93-6.90 (4H)	7.02-6.99 (8H)	4.44 (8H)	7.57 (5H)[b]	-
**TBD(1):BA(4) + CO2**	2.47 (4H)	2.36 (4H)	1.08 (4H)	7.14-7.10 (8H)	6.94-6.90 (4H)	7.01-6.98 (8H)	4.44 (6H)	-	4.81 (2H)

[a] Includes all exchangeable protons coming from both TBD (1H) and BA(1H) [b] Includes all exchangeable protons coming from both TBD (1H) and BA(4H)

Table S3: Chemical shifts obtained from the ^{1}H NMR spectra of the individual components – e.g. DBU and EG, the DESs resulting after their mixture in 1:1 and 1:4 molar ratios – e.g. DBU(1):EG(1) and DBU(1):EG(4), and the same DESs after loading with CO_2.

<table>
<tr><th rowspan="2">-</th><th colspan="8">2,3,4,6,7,8,9,10-octahydropyrimido[1,2-a]azepine (DBU)</th><th colspan="4">1,2 Ethanediol (EG)</th></tr>
<tr><th>H at C2 (CH_2)</th><th>H at C4 (CH_2)</th><th>H at C6 (CH_2)</th><th>H at C10 (CH_2)</th><th>H at C3 (CH_2)</th><th>H at C9 (CH_2)</th><th>H at C8 (CH_2)</th><th>H at C7 (CH_2)</th><th>H at C1&C2 2×(CH_2)</th><th>H at OH</th><th>H at C1* (CH_2-OCOO-)</th><th>H at C2* (CH_2-OH)</th></tr>
<tr><td>DBU</td><td colspan="2">3.38 (4H)</td><td>3.33 (2H)</td><td>2.49 (2H)</td><td>1.88 (2H)</td><td colspan="3">1.73 (6H)</td><td>--</td><td>--</td><td>--</td><td>--</td></tr>
<tr><td>EG</td><td>--</td><td>--</td><td>--</td><td>--</td><td>--</td><td>--</td><td>--</td><td>--</td><td>3.78 (4H)</td><td>5.43 (2H)</td><td>--</td><td>--</td></tr>
<tr><td>DBU(1):EG(1)</td><td colspan="2">3.38 (4H)</td><td>3.29 (2H)</td><td>2.49 (2H)</td><td>1.88 (2H)</td><td>1.78 (2H)</td><td colspan="2">1.71 (4H)</td><td>3.63 (4H)</td><td>6.38 (2H)</td><td>--</td><td></td></tr>
<tr><td>DBU(1):EG(1) + CO_2</td><td colspan="3">3.36 (6H)</td><td>2.64 (2H)</td><td>1.93 (2H)</td><td colspan="2">1.72 (4H)</td><td>1.65 (2H)</td><td>3.68 (2.8)</td><td></td><td>4.07 (0.6 H)</td><td>3.77 (0.6 H)</td></tr>
<tr><td>DBU(1):EG(4)</td><td colspan="2">3.43 (4H)</td><td>3.31 (4H)</td><td>2.53 (2H)</td><td>1.92 (2H)</td><td>1.79 (2H)</td><td colspan="2">1.72 (4H)</td><td>3.70 (16H)</td><td>6.02 (8H)</td><td>--</td><td>--</td></tr>
<tr><td>DBU(1):EG(4) + CO_2</td><td colspan="2">3.68 (4H)</td><td>3.41 (2H)</td><td>2.79 (2H)</td><td>2.05 (2H)</td><td>1.78 (2H)</td><td colspan="2">1.73 (4H)</td><td>3.61 (14H)</td><td>--</td><td>3.93 (2H)</td><td>--</td></tr>
</table>

Table S4: Chemical shifts obtained from the ^{1}H NMR spectra of the individual components – e.g. DBU and NMDEA, the DES resulting after their mixture in a 1:2 molar ratio – e.g. DBU(1): MDEA(2), and the same DES after loading with CO_2.

**Sample**	**2,3,4,6,7,8,9,10-octahydropyrimido[1,2-*a*]azepine (DBU)**								**2,2'-(Methylimino)diethanol (MDEA)**				
	**H** at C2 (CH_2)	**H** at C4 (CH_2)	**H** at C6 (CH_2)	**H** at C10 (CH_2)	**H** at C3 (CH_2)	**H** at C9 (CH_2)	**H** at C8 (CH_2)	**H** at C7 (CH_2)	**H** at C1&C1' 2×(CH_2)	**H** at C2&C2'&C2* 2×(CH_2)	**H** at C3 (CH_2)	**H** at OH	**H** at C1* (-CH_2-OCOO-)
**DBU**	3.38 (4H)		3.33 (2H)	2.49 (2H)	1.88 (2H)	1.73 (6H)			--	--	--	--	--
**NMDEA**	--	--	--	--	--	--	--	--	3.77 (4H)	2.70 (4H)	2.44 (3H)	5.38 (2H)	--
**DBU(1):NMDEA(2)**	3.40 (4H)		3.32 (2H)	2.51 (2H)	1.99 (2H)	1.77 (6H)			3.71 (8H)	2.66 (8H)	2.42 (6H)	5.87 (4H)	--
**DBU(1):NMDEA(2) + CO_2**	3.63 (4H)		3.39 (2H)	2.79 (2H)	2.02 (2H)	1.73 (6H)			3.63 (7H)	2.58 (8H)	2.34 (6H)	6.62 (4H)	3.96 (1H)

Table S5: Chemical shifts obtained from the ^{1}H NMR spectra of the individual components – e.g. TBD and EG, the DESs resulting after their mixture in 1:1 and 1:4 molar ratios – e.g. TBD(1):EG(1) and TBD(1):EG(4), and the same DESs after loading with CO_2.

**Sample**	**2,3,4,6,7,8-Hexahydro-1H-pyrimido[1,2-a]pyrimidine (TBD)**			**1,2 Ethanediol (EG)**			
	**H** at C2&C8 (CH_2)	**H** at C4&C6 (CH_2)	**H** at C3&C5 (CH_2)	**H** at C1&C2 2×(CH_2)	**H** at OH	**H** at C1* (CH_2-OCOO-)	**H** at C1* (CH_2-OH)
**TBD**	3.28 (8H)		1.96 (4H)	--	--	--	--
**EG**	--	--	--	3.78 (4H)	5.43 (2H)	--	--
**TBD(1):EG(1)**	3.24 (8H)		1.88 (4H)	3.56 (4H)	6.88 (3H)	--	--
**TBD(1):EG(1) + CO2**	3.17 (8H)		1.86 (4H)	3.49 (3H)	7.48 (3H)	3.84 (1H)	--
**TBD(1):EG(4)**	3.31 (4H)	3.25 (4H)	1.96 (4H)	3.62 (16H)	6.85 (8H)	--	--
**TBD(1):EG(4) + CO2**	3.39 (4H)	3.30 (4H)	2.00 (4H)	3.61 (12H)	5.84 (4H)	3.94 (2H)	3.69 (2H)

Table S6: Chemical shifts obtained from the ^{1}H NMR spectra of the individual components – e.g. TBD and MDEA, the DESs resulting after their mixture in a 1:2 molar ratio – e.g. TBD(1):MDEA(2), and the same DES after loading with CO_2.

**Sample**	**2,3,4,6,7,8-Hexahydro-1H-pyrimido[1,2-a]pyrimidine (TBD)**			**2,2'-(Methylimino)diethanol (MDEA)**				
	**H** at C2&C8 (CH_2)	**H** at C4&C6 (CH_2)	**H** at C3&C5 (CH_2)	**H** at C1&C1' 2×(CH_2)	**H** at C2&C2'&C2* 2×(CH_2)	**H** at C3 (CH_2)	**H** at OH	**H** at C1* (-CH_2-OCOO-)
**TBD**	3.28 (8H)		1.96 (4H)	--	--	--	--	--
**NMDEA**	--	--	--	3.77 (4H)	2.70 (4H)	2.44 (3H)	5.38 (2H)	--
**TBD(1):NMDEA(2)**	3.24 (8H)		1.98 (4H)	3.68 (8H)	2.61 (8H)	2.37 (6H)	6.33 (5H)	--
**TBD(1):NMDEA(2) + CO2**	3.38 (4H)	3.26 (4H)	2.00 (4H)	3.61 (7H)	2.55 (8H)	2.31 (6H)	7.16 (4H)	3.94 (1H)

Table S7: Chemical shifts obtained from the ^{13}C NMR spectra of the individual components – e.g. DBU and BA, the DESs resulting after their mixture in 1:1 and 1:4 molar ratios – e.g. DBU(1):BA(1) and DBU(1):BA(4), and the same DESs after loading with CO_2.

Sample	2,3,4,6,7,8,9,10-octahydropyrimido[1,2-*a*]azepine (DBU)									Benzyl Alcohol (BA)										
	**C1**	**C4**	**C6**	**C2**	**C10**	**C9**	**C7**	**C8**	**C3**	**C1**	**C3&C5**	**C4**	**C2&C6**	**CH_2**	**C at CO_2**	**C1***	**C3*&C5***	**C4***	**C2*&C6***	**CH_2***
**DBU**	159.52	52.59	48.37	44.56	37.21	30.05	29.07	26.64	23.29	-	-	-	-	-	-	-	-	-	-	-
**BA**	-	-	-	-	-	-	-	-	-	140.88	128.12	127.03	126.78	63.96	-	-	-	-	-	-
**DBU(1): BA(1)**	161.41	52.33	47.96	43.43	35.89	29.55	28.37	25.98	22.39	144.03	127.29	126.32	126.22	63.11	-	-	-	-	-	-
**DBU(1): BA(1) + CO_2**	163.78	53.46	48.47	41.15	34.65	29.44	27.79	25.07	21.11	141.96	128.28	127.04	126.87	64.42	158.60	139.68	127.97	126.77	127.04	66.94
**DBU(1): BA(4)**	162.33	52.09	47.56	42.36	35.04	29.11	27.73	25.33	21.61	142.14	128.03	126.69	126.63	63.63	-	-	-	-	-	-
**DBU(1): BA(4) + CO_2**	165.08	53.23	47.52	37.66	31.57	28.29	25.95	23.43	18.76	142.66	128.02	127.29	126.63	63.59	158.25	139.47	128.11	127.05	126.58	66.68

Table S8: Chemical shifts obtained from the ^{13}C NMR spectra of the individual components – e.g. TBD and BA, the DESs resulting after their mixture in 1:1 and 1:4 molar ratios – e.g. TBD(1):BA(1) and TBD(1):BA(4), and the same DESs after loading with CO_2.

Sample	2,3,4,6,7,8-Hexahydro-1H-pyrimido[1,2-a]pyrimidine (TBD)				Benzyl Alcohol (BA)										
	C10	C2&C8	C4&C6	C3&C7	C1	C3&C5	C4	C2&C6	CH_2	C at CO_2	C1*	C3*&C5*	C4*	C2*&C6*	CH_2*
**TBD**	151.53	46.81	37.91	20.73	-	-	-	-	-	-	-	-	-	-	-
**BA**	-	-	-	-	140.88	128.12	127.03	126.78	63.96	-	-	-	-	-	-
**TBD(1):BA(1)**	151.45	47.52	40.95	22.88	143.79	127.92	126.30	126.45	63.19	-	-	-	-	-	-
**TBD(1):BA(1) + CO2**	151.38	46.87	37.60	20.91	141.40	128.40	127.32	126.96	64.87	159.61	139.16	128.03	126.87	127.38	67.11
**TBD(1):BA(4)**	151.02	46.58	39.07	21.42	143.12	127.94	126.44	126.66	63.67	-	-	-	-	-	-
**TBD(1):BA(4) + CO2**	150.40	45.84	37.19	20.04	142.38	128	126.61	126.58	63.63	158.75	138.93	128.14	127.14	127.33	66.92

Table S9: Chemical shifts obtained from the ^{13}C NMR spectra of the individual components – e.g. DBU and EG, the DESs resulting after their mixture in 1:1 and 1:4 molar ratios – e.g. DBU(1):EG(1) and DBU(1):EG(4), and the same DESs after loading with CO_2.

Sample	2,3,4,6,7,8,9,10-octahydropyrimido[1,2-a]azepine (DBU)									1,2 Ethanediol (EG)			
	C1	C4	C6	C2	C10	C9	C7	C8	C3	C1&C2	C at CO2	C1*	C2*
**DBU**	159.52	52.59	48.37	44.56	37.21	30.05	29.07	26.64	23.29	--	--	--	--
**EG**	--	--	--	--	--	--	--	--	--	63.30	--	--	--
**DBU(1):EG(1)**	162.17	52.59	48.21	43.37	35.79	29.70	28.52	26.11	22.43	63.33	--	--	--
**DBU(1):EG(1) + CO_2**	164.38	53.66	48.53	40.66	34.29	29.36	27.60	24.86	20.80	63.89	159.56	67.23	63.12
**DBU(1):EG(4)**	163.40	52.81	48.19	42.37	35.10	29.48	28.05	25.15	21.79	63.35	--	--	--
**DBU(1):EG(4) + CO_2**	165.99	53.99	48.40	38.34	32.23	28.81	26.47	23.95	19.43	63.39	158.85	66.87	60.98

Table S10: Chemical shifts obtained from the ^{13}C NMR spectra of the individual components – e.g. DBU and NMDEA, the DES resulting after their mixture in a 1:2 molar ratio – e.g. DBU(1):MDEA(2), and the same DES after loading with CO_2.

**Sample**	**2,3,4,6,7,8,9,10-octahydropyrimido[1,2-*a*]azepine (DBU)**									**2,2'-(Methylimino)diethanol (MDEA)**					
	**C1**	**C4**	**C6**	**C2**	**C10**	**C9**	**C7**	**C8**	**C3**	**C1&C1'**	**C2&C2'**	**C3**	**C at CO_2**	**C1***	**C2***
**DBU**	159.52	52.59	48.37	44.56	37.21	30.05	29.07	26.64	23.29	--	--	--	--	--	--
**NMDEA**	--	--	--	--	--	--	--	--	--	60.05	59.26	42.75	--	--	--
**DBU(1):NMDEA(2)**	162.23	52.63	48.24	43.34	35.78	29.74	25.52	26.14	22.45	60.46	59.30	42.98	--	--	--
**DBU(1):NMDEA(2) + CO_2**	165.04	53.52	48.36	39.42	32.69	29.06	27.14	24.69	20.31	60.41	59.41	43.10	158	62.76	57.68

Table S11: Chemical shifts obtained from the ^{13}C NMR spectra of the individual components – e.g. TBD and EG, the DESs resulting after their mixture in 1:1 and 1:4 molar ratios – e.g. TBD(1):EG(1) and TBD(1):EG(4), and the same DESs after loading with CO_2.

**Sample**	**2,3,4,6,7,8-Hexahydro-1H-pyrimido [1,2-a]pyrimidine (TBD)**				**1,2 Ethanediol (EG)**			
	**C10**	**C2&C8**	**C4&C6**	**C3&C7**	**C1&C2**	**C at CO2**	**C1***	**C2***
**TBD**	151.53	46.81	37.91	20.73	--	--	--	--
**EG**	--	--	--	--	63.30	--	--	--
**TBD(1):EG(1)**	151.87	47.75	40.78	22.97	63.40	--	--	--
**TBD(1):EG(1) + CO2**	151.61	47.17	39.46	22.12	63.47	158.66	66.97	60.68
**TBD(1):EG(4)**	151.71	47.17	39.18	21.73	63.52	--	--	--
**TBD(1):EG(4) + CO2**	151.13	46.67	37.99	20.73	63.33	158.99	67.02	60.89

Table S12: Chemical shifts obtained from the ^{13}C NMR spectra of the individual components – e.g. TBD and NMDEA, the DES resulting after their mixture in a 1:2 molar ratio – e.g. TBD(1):MDEA(2), and the same DES after loading with CO_2.

**Sample**	**2,3,4,6,7,8-Hexahydro-1H-pyrimido [1,2-a]pyrimidine (TBD)**				**2,2'-(Methylimino)diethanol (MDEA)**					
	**C10**	**C2 & C8**	**C4 & C6**	**C3 & C7**	**C1&C1’**	**C2&C2’**	**C3**	**C at CO_2**	**C1***	**C2***
**TBD**	151.53	46.81	37.91	20.73	--	--	--	--	--	--
**NMDEA**	--	--	--	--	60.05	59.26	42.75	--	--	--
**TBD(1):NMDEA(2)**	151.92	47.75	40.78	22.96	60.58	59.13	43.08	--	--	--
**TBD(1):NMDEA(2) + CO_2**	151.38	46.65	37.81	21.17	60.31	59.36	43.02	158.44	62.88	57.49
